# The role of bacterial urease activity on the uniformity of carbonate precipitation profiles of bio-treated coarse sand specimens

**DOI:** 10.1038/s41598-021-85712-6

**Published:** 2021-03-17

**Authors:** Charalampos Konstantinou, Yuze Wang, Giovanna Biscontin, Kenichi Soga

**Affiliations:** 1 Engineering Department, University of Cambridge, Cambridge, CB2 1PZ UK; 2 Department of Ocean Science and Engineering, Southern University of Science and Technology, Southern Marine Science and Engineering Laboratory (Guangzhou), Shenzhen, 518055 China; 3 Engineering Department, University of Cambridge, Cambridge, CB2 1PZ UK; 4 Department of Civil and Environmental Engineering, University of California, Berkeley, CA 94720 USA

**Keywords:** Biological techniques, Biotechnology, Biogeochemistry, Environmental sciences, Engineering, Materials science

## Abstract

Protocols for microbially induced carbonate precipitation (MICP) have been extensively studied in the literature to optimise the process with regard to the amount of injected chemicals, the ratio of urea to calcium chloride, the method of injection and injection intervals, and the population of the bacteria, usually using fine- to medium-grained poorly graded sands. This study assesses the effect of varying urease activities, which have not been studied systematically, and population densities of the bacteria on the uniformity of cementation in very coarse sands (considered poor candidates for treatment). A procedure for producing bacteria with the desired urease activities was developed and qPCR tests were conducted to measure the counts of the RNA of the Ure-C genes. Sand biocementaton experiments followed, showing that slower rates of MICP reactions promote more effective and uniform cementation. Lowering urease activity, in particular, results in progressively more uniformly cemented samples and it is proven to be effective enough when its value is less than 10 mmol/L/h. The work presented highlights the importance of urease activity in controlling the quality and quantity of calcium carbonate cements.

## Introduction

Microbially induced carbonate precipitation (MICP) is an emerging and promising bio-cementation method that builds up calcium carbonate cementation around sand particles. The bonded material has increased strength and stiffness^1,2^. MICP has many potential applications in the field of geotechnical and structural engineering, including slope stabilisation^3,4^, liquefaction control^5,6^, mitigation of internal and surface erosion^7,8^, structural stabilisation^9–11^, bio-remediation^12,13^, to promote self-healing of soils or cracks, and bioconcrete^14–16^. The method can be applied successfully in a non-disruptive manner for various engineering applications at ambient temperatures and over large volumes. The MICP procedure has been also utilised recently to generate artificial specimens of weakly cemented carbonate sandstones^17^.

MICP by urea hydrolysis is one of the most efficient ways to implement the method and is induced by a series of reactions driven by urease^14^. Urea hydrolysis is an irreversible reaction producing ammonium and carbonate ions based on the following chemical equation:

$$\begin{aligned} \hbox {CO(NH}_{2})_{2} +2\hbox {H}_{2}\hbox {O} \rightarrow \hbox {2NH}_{4}^{+} + \hbox {CO}_{3}^{2-} \end{aligned}$$

The reactants raise both the alkalinity of the solution and the concentration of dissolved inorganic carbon (DIC)^18^, leading to an increase of the concentration of carbonate ions based on the chemical speciation of carbonic acid curves, as reported by Crittenden et al.^19^. In the presence of calcium ions, calcium carbonate precipitates into a solid form^18^ as follows:

$$\begin{aligned} \hbox {Ca}^{2+} + \hbox {CO}_{3}^{2-} \rightarrow \hbox {CaCO}_{3} \end{aligned}$$

*Sporosarcina pasteurii* is considered one of the best candidates for MICP among the many urease-producing strains studied in the literature, as its well-defined urease-synthesis behaviour is not repressed by ammonium and has no known pathogenicity^1^.

The positively charged calcium ions (cations) are attracted to the negatively charged bacterial cell walls^20^. Once urea is introduced in the bacteria solution, ammonium and dissolved organic carbon are released around the bacteria based on the first chemical equation presented. The alkaline environment and the local supersaturation around the cells, induced by the presence of calcium ions, result in calcium carbonate ($CaCO_{3}$) precipitation into solid form. As these processes continue, the cells become encapsulated, blocking the transfer of nutrients, and thus causing cell death^18^. Alternatively, once the targeted amount of calcite is achieved, nutrition can be cut off and the microbial cells will die. The precipitation of calcium carbonate involves several complex and interrelated processes of nucleation, transformation, and crystal growth^18,20,21^. Different forms of calcium carbonate (e.g. rhomboidal calcite or amorphous calcium carbonate, vaterite etc.) may also emerge, which can include transformable, unstable, and stable forms depending on the conditions of the reaction^21^.

Most of the biocementation studies previously conducted with MICP treatment have focused on optimising the recipe by altering the bio-chemical parameters (type of injection, flow rate, concentration of chemicals, ratio of chemical concentrations, density of bacterial population)^22–27^ however, more recently, the base material’s intrinsic properties have been receiving increased attention^28–30^. Careful adjustment of both the bio-chemical parameters and the properties of the base material are needed to meet the requirements of each application.

The majority of studies make use of poorly graded fine sands (sands with a narrow particle size distribution) with mean particle diameters of 0.15 to 0.7 mm^17,31–36^. Fine- to medium-grained sands allow for more flexibility in selecting the bio-chemical parameters of an MICP protocol as the bacterial and pore network sizes are more compatible. Mitchell and Santamarina^37^ produced a particle size compatibility relationship diagram indicating the relative dimensional boundaries of compatibility, according to which very fine or very coarse soils are not recommended for bio-cementation as the soil pores are smaller or larger compared to the size of the bacteria. Few studies have, therefore, focused on coarse sand^38^ and thus this material remains a testing-ground for the effectiveness of the MICP procedure in less than optimum conditions.

The effectiveness of the recipe is usually assessed via two metrics: chemical efficiency (amount of reactants converted into products) and uniformity of the final products. Research has shown that allowing enough time for complete conversion of the reactants into products is key^24,25^. This can be achieved through careful selection of bio-chemical parameters (bacterial optical density, urease activity, chemical concentrations, injection method) to produce slower MICP reactions in order to achieve more effective and uniform cementation.

Another parameter that can be adjusted to achieve more uniform bio-cemented sands is the injection method. Percolation is the most successful method as the flow path becomes self-adjusting allowing the introduction of the highest possible flow rates without dislocating the grains^7,39,40^. High chemical efficiency is obtained when injections are given at set time intervals with high flow rates, allowing reagents to reach deeper into a soil column^26,31,41^. High concentrations of bacteria should be avoided as they lead to clogging of the pores, preventing free flow, while enough time should be allowed between the injection of the bacteria and the injection of the first cementation solution to optimise bacteria settlement^42,43^. A urea to calcium ratio greater than one is recommended for effective recipe formulations, as it is proven to enhance the ureolysis and calcium carbonate precipitation rates^26,31^. Low concentrations of chemicals, or long retention times between subsequent injections, are needed for the full chemical reactions to take place throughout the specimens, even in the remotest places where bacteria concentration is low^21,24,43^. Longer retention times increase the overall duration of the treatment, causing decline of bacterial activity^24^, which can also contribute to more uniform bio-cementation^39^.

The issue of uniformity has been extensively studied, with the literature showing contradictory results. Although repeatability and uniformity should be considered independently, the two are quite often combined in the literature because inhomogeneous specimens are also likely to produce inconsistent characteristics, and hence poorly reproducible results. Uniformity decreased as the degree of cementation increased in the experiments by Feng et al.^34^ and Lin et al.^44^, while Cui et al.^36^ showed that uniformity increased as the cementation level increased. Dawoud^33^ found higher calcite deposition around the injection point, but very small variance in the rest of the specimen, showing rather uniform samples.

Based on the findings in the literature, it follows that the selection of bacteria with lower urease activity as part of an MICP procedure with low concentration of cementation solution compared to the rate of flow with gravity injections, would provide a balance of slower MICP reactions, promoting uniform and repeatable products. However, the bacterial urease activity as part of an effective MICP protocol has not been examined previously in a systematic way. Cheng et al.^35^ report that lower urease activity resulted in more efficient bonding between the particles and thus higher unconfined compressive strength (UCS), but did not assess uniformity of cementation nor effectiveness of the process. The lack of research on the effects of urease activity on MICP treatments can be partially explained by the difficulty of controlling the urease enzyme levels across bacteria cultivated under similar conditions. Whiffin^1^ reported great enzyme variability with a factor of more than ten, suggesting that the cultivation conditions conducive to growth do not guarantee consistent urease production.

This study first presents a procedure for controlled preparation of bacteria with the desired urease activity levels demonstrating consistency by characterising and quantifying the RNA of the Ure-C subunit of the urease enzyme via quantitative polymerase chain reaction (qPCR) tests. Once the procedure is established, the role of urease activity on the bio-cementation of very coarse granular materials, typically considered poor candidates for successful MICP treatment, is examined, demonstrating that urease activity is an important parameter to consider when developing MICP protocols for the preparation of uniformly cemented specimens. By slowing the MICP bio-chemical reactions, within a denser bacterial population, more efficient formation and distribution of carbonate across the specimens is expected. For these experiments, a pure culture of *Sporosarcina pasteurii* was used in the experiment with an initial inoculation of bacteria followed by fifteen chemical solution injections introduced at 24 h intervals.

## A study for preparation of bacteria with the desired urease activity

This section presents a method to successfully control the urease activity of *S. pasteurii*. A urea solution of $18\, \hbox {mL}$ with a concentration of 1.11 M was inoculated with 2  mL of bacteria, as this is common practice^41^. The method followed for the quantification of urease activity is presented in detail in the “Methods” section.

**Figure 1:**
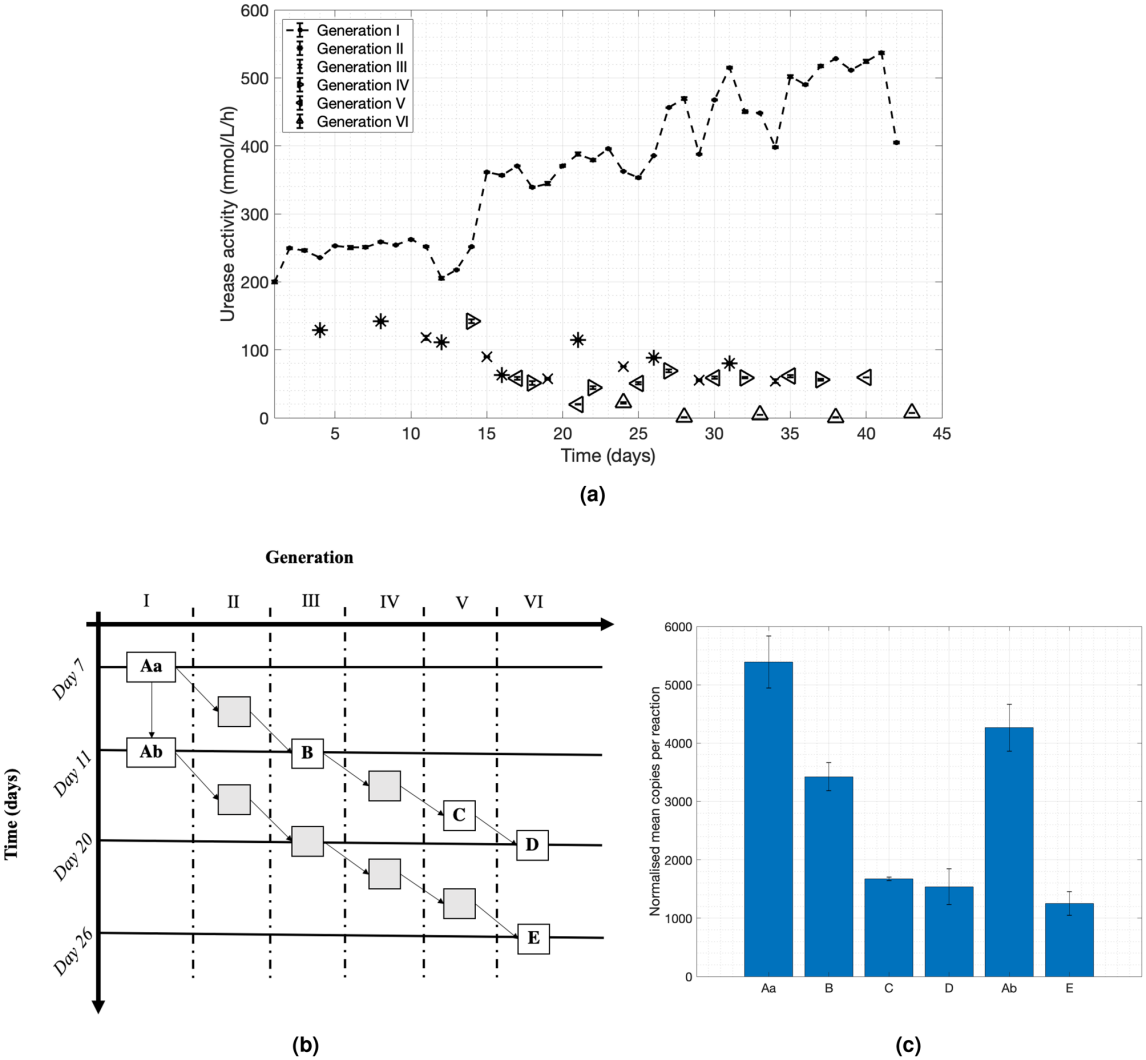
(**a**) A study for delivering bacteria of a desired urease activity. The urease activity is measured in mmol/L/h. Bacteria were first extracted from the petri dish and introduced to the nutrient broth liquid (NBL). These bacteria are referred to as ‘Generation I’ and the urease activity was monitored on a daily basis up until Day 42. Every 3–5 days 2 mL of bacterial suspension were introduced into 300 mL of new nutrient broth liquid and placed in incubation to reach an $OD_{600}$ of 1. The urease activity of the new bacteria (Generation II) was also monitored on Day 1 and 3. On Day 3, 2 mL from the Generation II bacteria were introduced to 300 mL of new nutrient broth and placed in incubation to reach an $OD_{600}$ of 1 to grow Generation III. The process was repeated in the same way until Day 42 and Generation VI; (**b**) Schematic view of the relations between samples taken at different phases of the experimental program for qPCR tests; (**c**) the qPCR test results from bacterial samples: normalised mean copies of the RNA of the Ure-C subunit.

Bacteria were first extracted from the petri dish and introduced to the nutrient broth liquid (NBL) which did not contain urea, as described in the “Methods” section. The mixture was stored at ${4}\, ^{\circ }\hbox {C}$. These bacteria are referred to as ‘Generation I’ in Fig. 1a and Fig. S1 (supplementary material). The urease activity of these bacteria was monitored on a daily basis up until Day 42. Every 3–5 days 2 mL of bacterial suspension were introduced into 300 mL of new nutrient broth liquid and placed in incubation to reach an $OD_{600}$ of 1. The urease activity of the new bacteria (Generation II) was also monitored on Day 1 and 3. On Day 3, 2 mL from the Generation II bacteria were introduced to 300 mL of new nutrient broth and placed in incubation to reach an $OD_{600}$ of 1 to grow Generation III. The process was repeated in the same way until Day 42 and Generation VI. The bacterial suspensions were stored at all times at ${4}\, ^{\circ }\hbox {C}$.

The bacterial urease activity decreased substantially from generation to generation. The generations of bacteria created from the first week’s Generation I bacteria experienced a steady reduction in urease activity, while the reduction for the samples generated on later phases declined even more rapidly to the lowest values. For example, the urease activity of Generation II bacteria declined faster than that of Generation I after the same amount of time in the fridge. The generation-to-generation decline was more pronounced and quicker compared to the response monitored from bacteria of younger generations created from ‘older’ Generation I bacteria. This decline of urease activity might be attributed to the adjustment of bacteria under these conditions to save energy as there was no urea in the culture medium.

As shown in the diagram of Fig. 1a, the urease activity of Generation I bacteria started to increase substantially after the second week of storage in the fridge, probably as a shock response. A recent study conducted by Ma et al.^45^ investigated the effects of urease activity specifically when adding urea to the culture medium. The authors demonstrated that the addition of the urea enhanced bacterial growth and showed faster urease production, although the maximal urease activity was lower compared to cultivation without urea. This explains why the urease activity increased in Generation I bacteria. While stored in the fridge, bacterial growth was considerably inhibited, but not totally disabled. At the end of the second week, bacteria were generated within the same culture medium without urea resulting in a delayed maximal increase of urease activity (from day 14 onwards).

The findings on the urease activities across the bacterial generations and across time were supported by quantitative polymerase chain reaction (qPCR) tests. *S. pasteurii* urease has a quaternary structure in the form of $\alpha _{3}\beta _{3}\gamma _{3}$ with three crystallographically related active sites, one in each sub-unit^46^. Of the three structural genes, Ure-A, Ure-B and Ure-C, Ure-C is reported to have the greatest molecular weight^46–48^. The qPCR tests targeted the quantification of the RNA copies of the Ure-C subunit against three reference genes to further explore the results obtained by the program for generating bacteria with a pre-defined urease activity and to assess whether this behaviour was consistent throughout the injection period in the MICP process. Further information on the qPCR tests is provided in the “Methods” section.

A lower RNA copies count of the Ure-C subunit indicates decreasing activity during the bio-cementation process and was not seen to reverse its course in these tests. Six bacterial samples were taken from several points in the program. Samples Aa and Ab were from Generation I on day 7 and 11, while sample B was from Generation III, as produced from Aa. Sample C was from Generation V and samples D, E from Generation VI. The schematic shown in Fig. 1b shows a simplified view of the relations between the samples.

There are significant differences in the numbers of RNA Ure-C copies counts across the samples (Fig. 1c), which agree with the measurements of the bacterial urease activity taken by utilising the standard protocol by Whiffin^1^. The reduction of the urease activity measured in the program could be linked to the fact that the Ure-C gene appears to be less expressed. The number of copies of the Ure-C gene transcription decline from generation to generation (Specimen Aa to B, C and D), while a smaller decline is observed after a time interval in the same generation (specimen Aa to Ab).

Both the measurements of activity of the populations and the Ure-C expression were consistently in agreement. The results establish that it is possible to control urease activity of the bacteria to a sufficient level for the purposes of this study. Bacteria can be produced based on the required specifications in a simple manner.

Once the issue of ‘manufacturing’ bacteria with the desired urease activity was resolved, bacterial populations with various urease activities were generated and introduced into very coarse sand samples. The next section examines the effects of bacterial urease activity on the bio-cementation process. Bacterial solutions with different levels of activity were prepared with a similar method to the one illustrated above, albeit less extensive.

## The role of urease activity on bio-cementation of coarse sand specimens

The second major objective of this study was to validate the hypothesis that a lower urease activity would produce more uniform and effective samples by promoting slower bio-chemical reactions as part of an MICP procedure. Various generations of bacterial suspensions were prepared following the ‘manufacturing’ process presented in the previous section with a declining trend in activity. These were utilised in flask experiments and in sand column experiments with very coarse sand samples. Such large particles were chosen to demonstrate the significance of the parameter under investigation through successful cementation of materials that are not considered ideal candidates for MICP treatment.

A preliminary study was conducted utilising bacterial suspensions with three urease activities (high - 294 mmol/L/h, medium - 66.14 mmol/L/h and low - 19.33 mmol/L/h) and populations with $OD_{600}=1$, 2 and 3 in flasks without sand particles and without effluent in triplicates. Then, coarse sand samples were generated in duplicates using bacteria with five urease activities (237.4, 112.9, 44.1, 7.5 and, 0.45 mmol/L/h) and populations at two different optical densities ($OD_{600}=2$ and 3) giving a total of 20 specimens (2 $\times$ 2 optical densities $\times$ 5 urease activities). The urease activity values reported throughout this paper correspond to the values measured following the protocol suggested by Whiffin^1^. The urease activities and specific urease activities measured following both the standard protocol by Whiffin^1^ and the protocol that simulates the conditions within the sand columns are reported in supplementary material. Both protocols are presented in detailed in the “Methods” section. The rest of the bio-chemical parameters remained fixed in order to isolate the effects of the bacterial parameters. The MICP recipe is described in the “Methods” section. Fifteen injections were administered to each sample in both the flask and coarse sand column experiments.

The precipitating calcium carbonate and the chemical efficiency were calculated in the conical flask experiments. The biocemented sand specimens were visually inspected once extracted from the molds to qualitatively characterize the effects of urease activity and quantitative analysis was conducted to assess uniformity and average calcium carbonate content. The effects of the bacterial population density in conjunction with the urease activity were also examined. MicroCT scanning and SEM imaging were used to measure the final porosity and assess morphological differences within the structure.

### Flask experiments results

This series of experiments was conducted to observe the effects of the various levels of urease activities without the presence of a granular medium, acting as a guide for the selection of combination of bacterial properties in the sand column experiments. The rest of the parameters and experimental conditions were selected to be the same between the flask and sand column experiments. As mentioned previously, fifteen injections of chemical solution were administered and were introduced at 24 h intervals.

**Figure 2:**
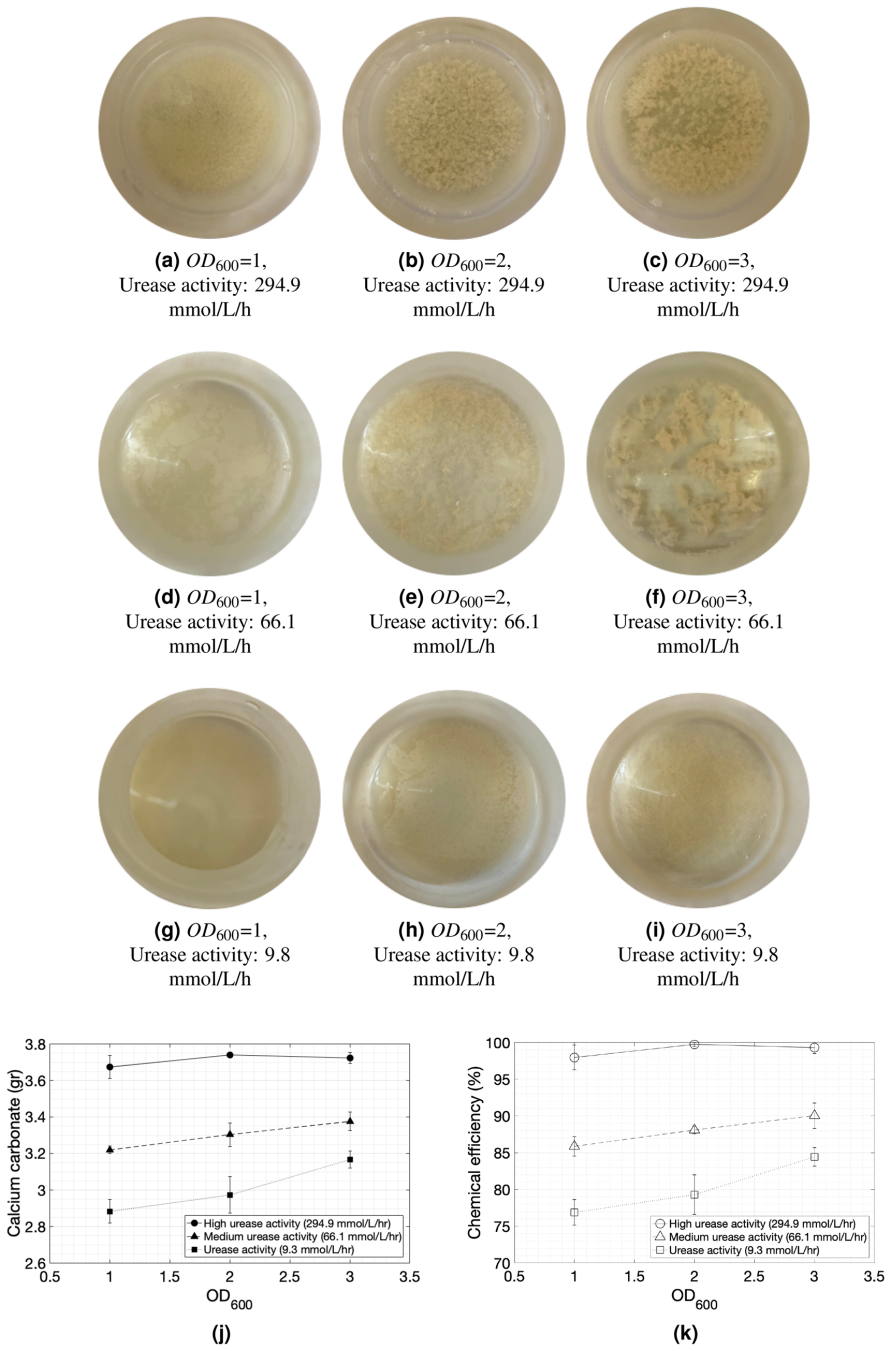
(**a**–**i**) Photographs of bacterial and cementation solution suspensions taken 24 h after the first cementation solution injection. From left to right, the initial optical density at $OD_{600}$, increases from 1 to 2 and 3. From top to bottom, the urease activity decreases. (**j**) The precipitated calcium carbonate concentrations (gr) and (**j**) chemical efficiency (%) of the flask experiments across the various optical densities and urease activities. The carbonate concentrations are shown as solid shapes and the efficiencies as hollow shapes. The urease activity values reported in the figure are measured with the standard protocol^1^.

After the first cementation solution injection, carbonate crystals started developing at a rate similar to the urea hydrolysis rate measured based on the protocol that is described in the “Methods” section. The precipitation rate depends on both the bacterial population and the urease activity. Denser bacterial and cementation solution aggregations appear for the combination of the highest bacterial population and highest urease activity as shown in the photographs in Fig. 2a–i.

In the subsequent cementation solution injections, the carbonate crystals started to precipitate at the bottom of the flask. The results for the high urease activity experiments are in agreement with the findings of the microfluidic experiments by Wang et al.^49^ who studied the effects of bacterial optical density. For low optical densities ($OD_{600}$ = 0.1), the authors reported that the crystals formed more slowly and were larger in size, but fewer. For very high optical densities ($OD_{600}$ = 3) precipitation increased, but large amounts of the unstable forms of calcium carbonate were observed.

These effects, however, were more evident in the flasks containing the bacteria with high urease activities while hardly any crystals were found to be suspended in the case of flasks containing bacteria of low urease activities. All cases, though, resulted in carbonate precipitation at the bottom of the flasks. As suggested by Wang et al.^49^, if enough time is given, small crystals tend to dissolve at the expense of the growth of larger carbonate crystals of the same type. The time window between two subsequent injections selected in this study was 24 h which was sufficiently wide to allow for this phenomenon to take place.

As the bacterial population increased, the precipitated carbonate mass and the chemical efficiency decreased (Fig. 2j,k), however the reaction rates and the efficiency seemed to be dictated by the urease activities. The lower the bacterial urease activity was, the lower the chemical efficiency was. The precipitation efficiency of the process was generally high because without the presence of a granular medium, the cations and anions were completely diffused throughout the flask.

The injection interval was selected to be 24 h in order to allow for the dissolution of smaller crystals to then be replaced by larger crystals as suggested by Wang et al.^49^. As seen in Fig. S6 (supplementary material), the amount of ammonium produced within a 24-h window, which tracks the hydrolysis rate, shows that the time given was not sufficient for the complete transformation of reactants into products. This observation was not evident in this series of experiments since there was no outflow, thus, the reactions continuously took take place in the conical flasks even with ’old’ chemical solutions from previous injections.

### Sand columns results

Based on the findings in the previous section, higher populations of bacteria at lower urease activity levels would essentially be the best candidates for uniform and effective cementation of very coarse particles. Larger populations of bacteria would increase the probability of attachment on the surface of the particles or pore throats and at the same time increase the amount of precipitating calcium carbonate. Therefore, larger optical densities of $OD_{600}$ 2 and 3 were selected for the subsequent experiments.

**Figure 3:**
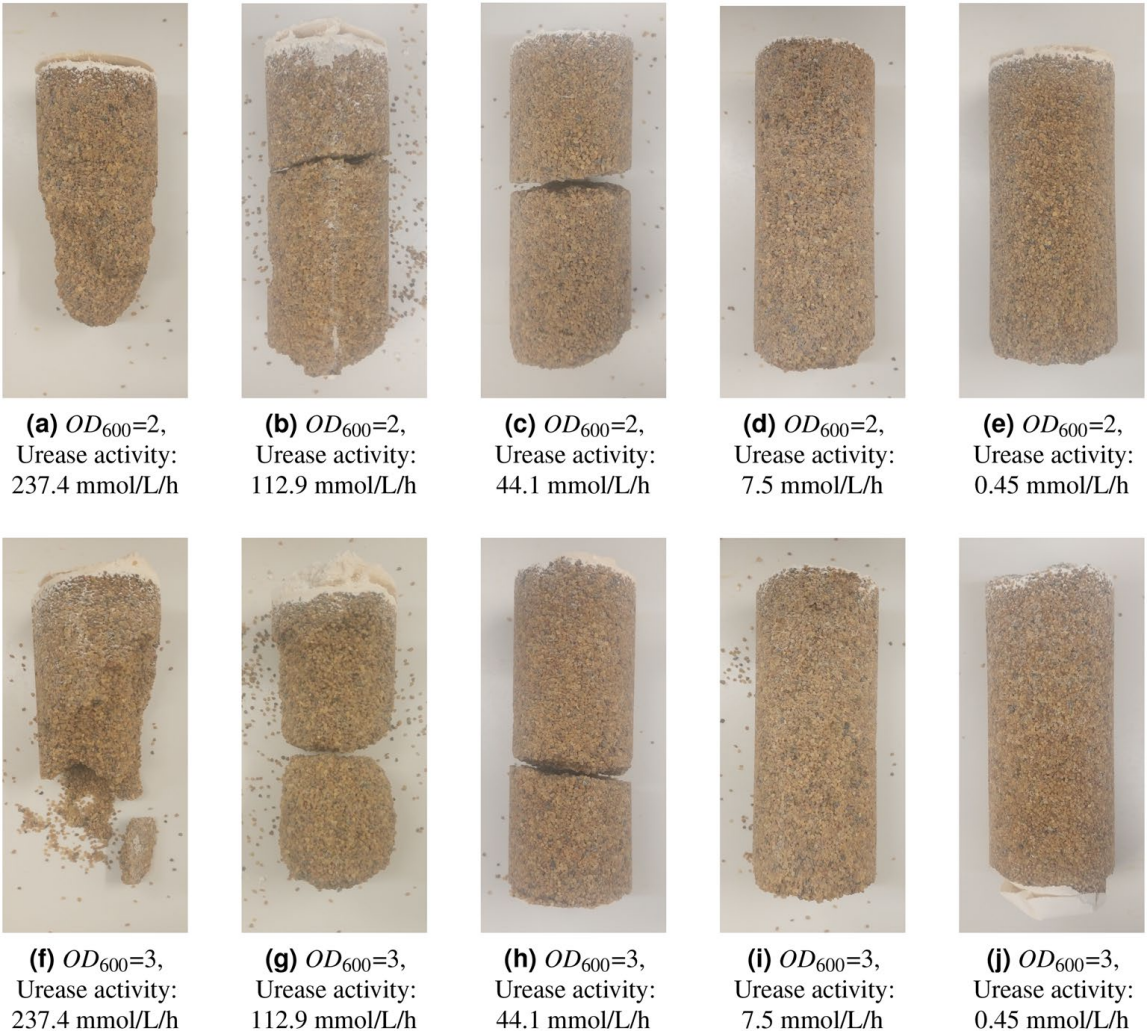
(**a**–**e**) Photographs of specimens taken with $OD_{600}$ = 2 and activity in descending order (237.4, 112.9, 44.1, 7.5 and, 0.45 mmol/L/h respectively, measured following the standard protocol^1^) (**f**–**j**) Photographs of specimens taken with $OD_{600}$ = 3 and activity in descending order as before. The injection was from top to bottom.

When high urease activity bacteria were used, the distribution of the cementation was highly uneven and specimens could not be extracted without breaking as seen in Fig. 3. These samples also exhibited a large variability of cementation across their height. The sample treated with the bacteria of the highest urease activity was only cemented in the first few centimetres below the injection point. Samples injected with medium urease activity bacteria were mostly uniformly cemented, but still presented areas with barely any cementation, resulting in disaggregation of the material during de-molding. Low urease activity bacteria produced the most uniform specimens.

At higher urease activities the chemicals were consumed quickly in the area near the injection point preventing them from reaching the more remote places away from the injection point. Once the urease activity decreased, the chemicals penetrated further into the samples and the reactions reached deeper through the soil column, confirming that allowing enough time for the reactions to take place even at the remotest places with the specimen is necessary.

Abiotic control experiments with injection of the cementation solution without bacterial injection were conducted in order to demonstrate that urea hydrolysis is indeed induced by the metabolic activity of *S. pasteurii*. As seen in the photograph in Fig. S3a, the specimen disaggregated at the grain scale during de-molding showing no evidence of cementation. Nil cementation was registered as shown in Fig. S3b,c. The blank experiments show that urea was hydrolysed only in the presence of the specific bacterial population, since the sand used in this work was supplied washed and dried, free of any organic components (inert).

**Figure 4:**
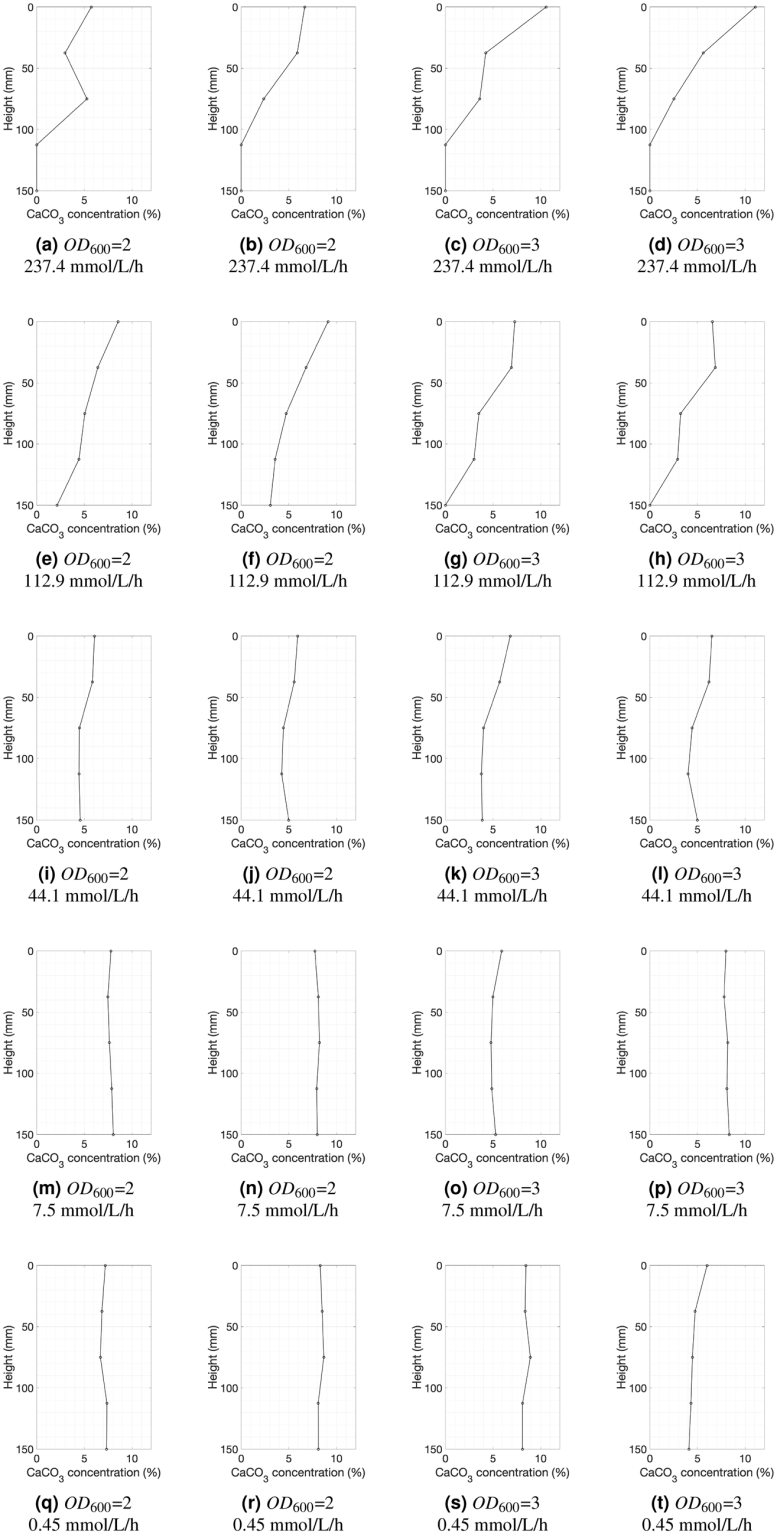
Calcium carbonate concentration profiles: each row reports results for a different urease activity with values decreasing from the largest activity at the top to the smallest at the bottom (237.4, 112.9, 44.1, 7.5 and, 0.45 mmol/L/h respectively, measured following the standard protocol^1^); the first two profiles in each row are for samples generated with $OD_{600}$ = 2, whilst the last two profiles of each row are for samples generated with $OD_{600}$ = 3. The calcium carbonate concentration of each specimen was measured at 5 points along its height and the injection was from top to bottom.

**Figure 5:**
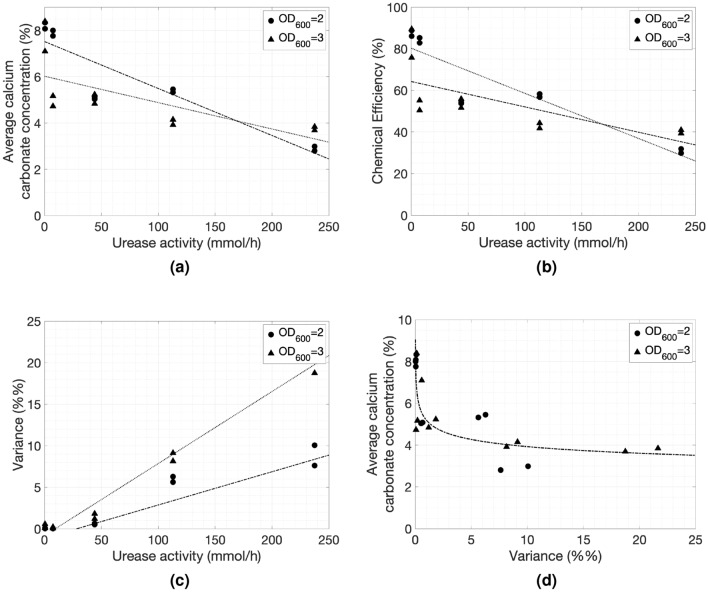
(**a**) Average calcium carbonate concentration (%) calculated as the average value of each calcium carbonate precipitation profile shown in Fig. 4, (**b**) Chemical efficiency (%) calculated as the amount of reactants converted intro products, (**c**) Variance (%%) of the five points of each precipitation profile with respect to urease activity measured following the standard protocol^1^. The dash-dotted line is the linear fit for specimens generated with bacterial populations at $OD_{600}$ = 2 and the dotted line is for specimens generated with bacterial populations at $OD_{600}$ = 3. (**d**) Average calcium carbonate concentration (%) with respect to Variance (%%) calculated for each precipitation profile.

The average calcium carbonate concentration was calculated based on the individual carbonate concentration profiles shown in Fig. 4, while the uniformity was assessed based on the variance of the precipitation profiles. Overall, the average calcium carbonate concentration in the specimens (Fig. 5a) decreased with the increase in urease activity. Lower optical densities resulted in higher cementation levels, although the trend reversed at high urease activity. The low average cementation level at high urease activity is a result of the uneven distribution of the calcium carbonate, which tended to be high at the top of the specimens and low or nil at the bottom. The two lines for bacteria with optical densities of $OD_{600}$ = 2 and $OD_{600}$ = 3, cross each other because when the bacterial population was higher and at 7.5 mmol/L/h/$OD_{600}$ the average calcium carbonate concentration was low compared to the specimens generated with the lower bacterial density solution for the same activity. This indicates that, when designing MICP protocols, one of the two parameters has to act as a limiting factor with very low values. This is also demonstrated by the chemical efficiency relationship with the urease activity (Fig. 5b) where at an $OD_{600}$ = 3 with the urease activity being at around 7.5 $mmol/L/h/OD_{600}$ the chemical efficiency was at about 50%. Generally, the chemical efficiency also decreased as the urease activity declined which was expected based on the findings of the flask experiments. This indicates that a large portion of the the reactants was removed with the effluent. However, a lower chemical efficiency is acceptable at the expense of more uniformly cemented specimens.

As stated previously, the variance of the carbonate concentration was used as the metric representing how uniform the specimens were. The variance of the carbonate concentration decreased as the urease activity decreased, as shown in Fig. 5c. Under 10 mmol/L/h/$OD_{600}$, the variance was very low and the samples became uniform. The effects of bacterial population density $OD_{600}$ on the uniformity of the specimens were clear at high urease activities, whereas the variance of carbonate concentration for $OD_{600}$ = 3 specimens was about twice of that of the specimens formed with the low bacterial population. This behaviour faded out as the bacterial urease activity decreased. Practically, the maximum difference in cementation level within each specimen was around 5–6% for high urease activities reducing to less than 1% in the range of the highest urease activities.

The MICP process was much more efficient at low urease activities, as all parameters, and specifically the combination of cementation level and variance, became much better once the activities were low enough (see Fig. 5d). With reference to the pre-injection urease activity measurements at a bacteria ($OD_{600}$ = 1) to urea (1.1 M) ratio of 1:9, the threshold value for producing uniform samples at bacterial populations with optical densities equal or higher than 2 seems to be under 10 mmol/h.

**Figure 6:**
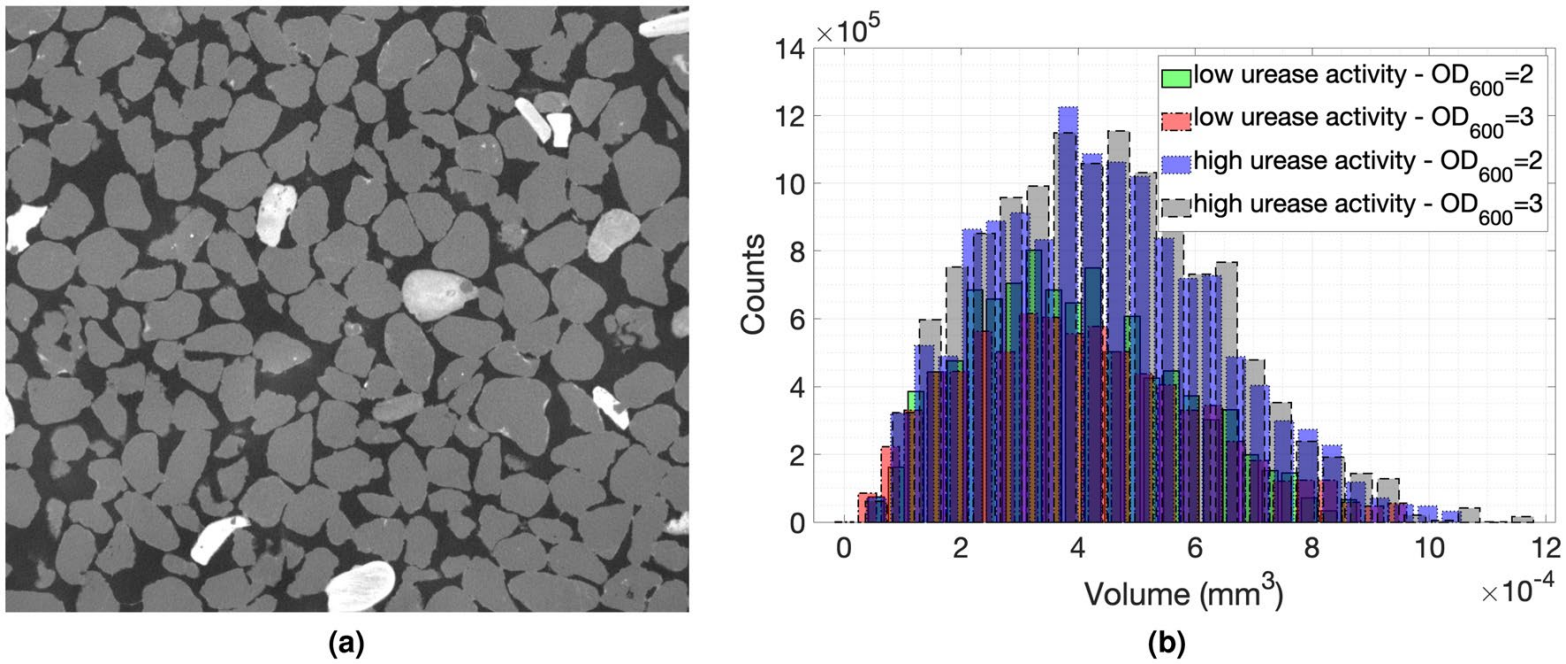
MicroCT imaging: (**a**) A slice of the specimen with its gray values showing the presence of iron minerals, (**b**) The histograms showing the pore network sizes of the specimens with the highest and lowest urease activities (237.4 and 0.45 mmol/L/h, respectively, measured following the standard protocol^1^) for bacterial populations at $OD_{600}$ = 2 and 3. Stacks of images were obtained and transferred to the ImageJ software^52,53^. The images were then binarised and were analysed via the BoneJ plugin^54^ to obtain the porosity and distribution of voids^55^.

The porosity and pore network configuration of the specimens generated with bacteria with the highest and lowest urease activities at the two population densities utilised in this work were examined with the aid of MicroCT tomography. Samples were obtained in the middle of the specimens. In cases where there was non-uniform cementation, samples were taken in the middle of the remaining intact portion of the specimen. The sand element analysis showed that it comprised 94.6% $SiO_2$, 4% $Fe_2O_3$ and less than 1% of $Al_2O_3$, $TO_2$, $K_2O$ and, $Na_2O$. The iron oxides are the white grains of larger density shown in the slice in Fig. 6a.

The initial porosity of the sand columns before any cementation was applied was 0.37 and after the cementation was applied the porosity reduced down to 0.29–0.31 for the two specimens at low urease activities while it reduced to about 0.34–0.35 for the specimens at high urease activities where hardly any cementation was observed. The distribution of the pores is shown in the histogram in Fig. 6b confirming that the specimens generated with bacteria at low urease activities had smaller porosity but also smaller-sized pores. More information about the procedure followed to calculate the porosity can be found in Fig. S4 in supplementary material.

**Figure 7:**
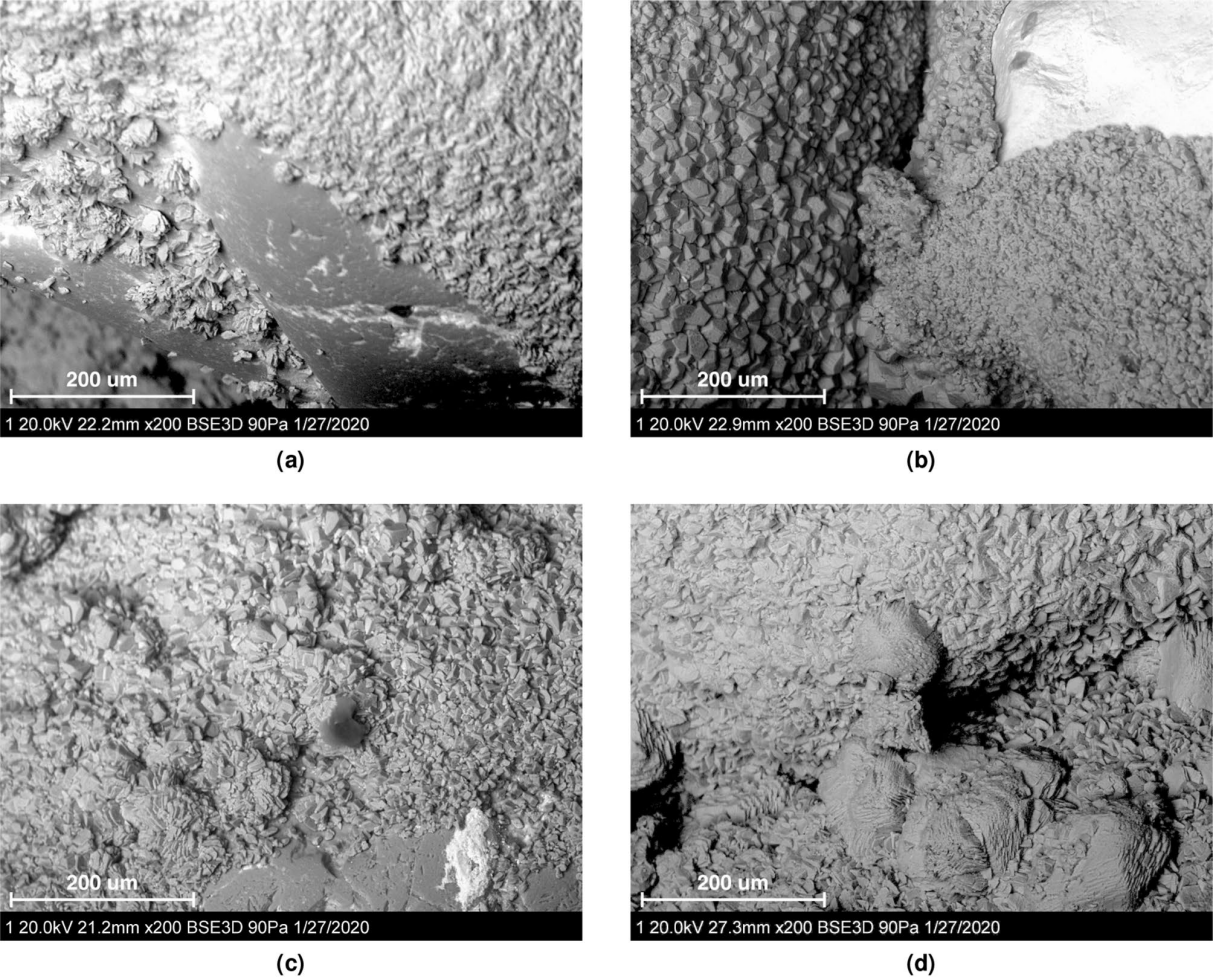
SEM imaging of the specimen generated with (**a**) the highest urease activity (237.4 mmol/L/h) at $OD_{600}$ = 2, (**b**) the highest urease activity (237.4 mmol/L/h) at $OD_{600}$ = 3, (**c**) the lowest urease activity (0.45 mmol/L/h) at $OD_{600}$ = 2 and, (**d**) the lowest urease activity (0.45 mmol/L/h) at $OD_{600}$ = 3.

The morphology of the specimens and microstructure were also examined. At higher urease activities the resulting carbonate crystals were of similar size, cubic in shape and evenly distributed on the surface of the grains (Figs. 7 and S5). As the activity decreased crystals became larger in size and formed clusters seemingly more effective at forming bridges between particles. The observations are more pronounced for bacterial populations of $OD_{600}$ = 3.

The results indicate that a combination of parameters for the MICP process needs to be selected such that a balance of slower MICP reactions is achieved. The key to obtaining uniform and repeatable products was to slow down the MICP reactions, by reducing concentration of the cementation solution, to allow full permeation of the specimens. The high optical density of the bacterial solution likely resulted in aggregation once the cementation solution was first introduced^43,50^, improving the retention of bacteria in the larger pore spaces. Long retention times were then required to ensure a high percentage of the cementation solution was transformed into calcium carbonate. While these MICP parameters have been investigated in previous studies, low bacterial urease activity has not been considered as a way to promote slower, but more uniform, reactions across the height of the specimen.

### Monitoring parameters of the MICP process

**Figure 8:**
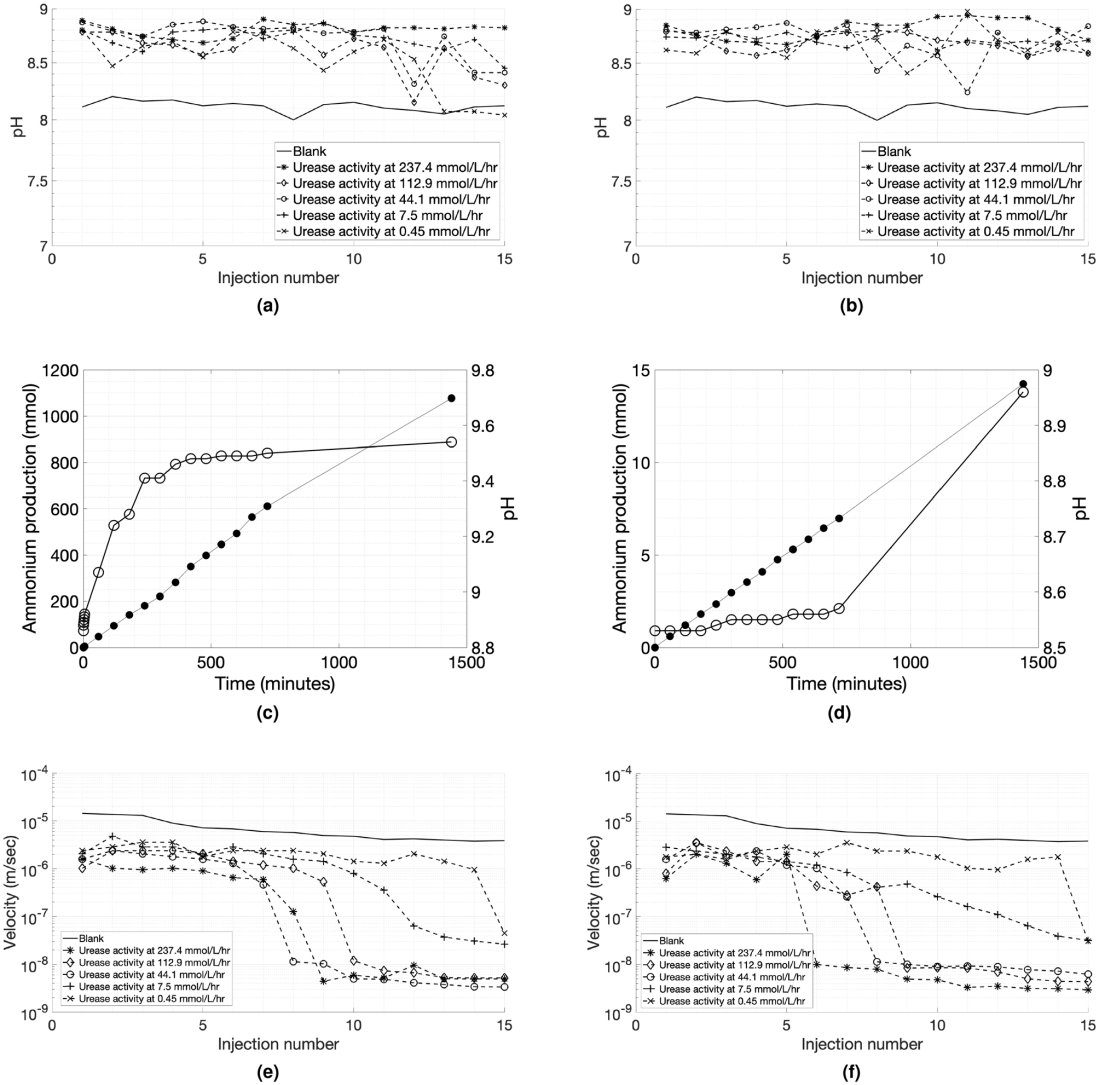
Effluent pH values across the injections during the bio-treatment events on a semi-log plot for (**a**) bacteria solutions at a population of $OD_{600}$ = 2 and (**b**) bacteria solutions at a population of $OD_{600}$ = 3. Ammonium production (mmol) measurements ($\bullet$) and pH measurements ($\circ$) within a time window of 24 h for the bacterial solutions utilised in the sand column experiments: (**c**) 237.4 and, (**d**) 0.45 mmol/L/h measured following the protocol by Whiffin^1^. (**e**,**f**) Percolation velocities across the injections during the bio-treatment events on a semi-log plot for (**e**) bacteria solutions at a population of $OD_{600}$ = 2 and (**f**) bacteria solutions at a population of $OD_{600}$ = 3.

The pH values of the culture solution were around 9.0 for all cases, without showing any considerable variations. The pH of the cementation solution before injection was 8.2, while the pH of its effluent was around 9.0 in the initial volume, reducing to 7.8-8.5 towards the last injection cycles. This indicates either that bacteria became less active towards the end of the treatment or that carbonates precipitated effectively (see Fig. 8a,b). However, almost all values remained above the pH values of the effluent of the blank experiment as shown in the diagram.

The $OD_{600}$ of the effluent was around 1.5 for columns injected with bacterial suspension of $OD_{600}$ = 2, while the $OD_{600}$ of the effluent was around 1.8-2.0 for columns injected with $OD_{600}$ = 3. These values remained constant across bacterial urease activities, indicating that the growth dynamics of the system did not change. The pH values of the bacterial effluent also remained unchanged at around 8.5-9.0.

The ammonium production curves of all bacterial suspensions were calculated within a time window of 24 hours following the standard protocol by Whiffin^1^ and the protocol that simulates the conditions within the sand columns (presented in the “Methods” section). Details can be found in the supplementary material in Figs. S6–S8.

The ammonium production curves for the bacteria with the highest and lowest urease activities within the sand columns were almost linear within the 24-h window, while the pH values escalated faster in the active bacterial suspensions, reaching a plateau with a value of about 9.53 (Fig. 8c,d) . In the case of the least active bacterial suspensions, the increase of the alkalinity is slower, because the ammonium production is much lower compared to the first case. The ammonium production curves show that for the low urease activities the reaction rates were not sufficient for the complete transformation of reactants into products within a 24-h window, which explains the lower chemical efficiencies observed. However, a lower chemical efficiency is acceptable at the gain of more uniformly cemented specimens.

The urease activity and the specific urease activity for each combination of bacterial population were compared to the input urease activity measured with the conventional method at a bacterial population of $OD_{600}$ = 1 for each specimen (more information can be found in the supplementary material in Fig. S9). The bacterial urease activity in the effluent was significantly different from the activity of the bacterial suspension before injection. These differences in the values though, remained consistent, since there was no cross-over across the decreasing order of activities measured before the injection. The activity measurements before and after the injection prove that indeed the pre-injection activity can be used as a reference value for MICP experiments.

The percolation time of each injection was defined as the time required for 1.1 pore volume (PV) of chemical solution to fully penetrate the specimen. The height of the soil divided by the percolation time gave a rate of injection or percolation velocity. Low percolation velocities could be interpreted as an indication of clogging, since a low flow rate is most likely caused by localised cementation at the inlet. The two bacteria solutions ranked with the lowest urease activities maintained the injection velocities even on the last injection while the percolation rate of bacterial solutions with higher activities seems to drop in the 6th, 7th and 8th injection for $OD_{600}$ = 3 (Fig. 8f) and a few injections later for $OD_{600}$ = 2 (Fig. 8e). Figure 4 shows that the specimens generated with bacteria of higher urease activities have steeper carbonate concentration gradient with the injection point having the highest cemantation level compared to the rest of the specimen. The average carbonate concentration also increases with decreasing urease activity. Therefore the drop was not due to reduction in porosity of the overall specimen, but due localised porosity reduction.

Injection percolation rates seem to give a simple indication of the uniformity of the specimens. The samples that broke or disaggregated during extraction or measured high variations of cementation level displayed a sudden decline in percolation rate at the 6th to 8th injection or even earlier for higher bacterial densities, which also resulted in larger variance. Urease activity is preferred over specific urease activity as an indicator because it better represents actual conversion rates.

## Conclusions

A simple, but effective, process is developed to control the variability of urease activity in bacterial populations used for bio-cementation and to deliver bacteria with the desired urease activity, irrespective of the bacteria. The bacterial urease activity consistently reduces from generation to generation. The same trend is observed with respect to the Ure-C RNA copies.

This type of ‘manufacturing’ process is potentially useful in applications such as remediation of contaminated sites with cement (reducing permeability) or for preventing material removal by seepage (internal erosion), and especially in cases were artificial specimens for laboratory testing are required. Each application requires different adjustment of the MICP procedure parameters and this procedure enables the selection of bacterial urease activity to provide another option to control the bio-cementation process.

The comparison of the bio-cemented specimens resulting from various combinations of urease activities and optical densities provided essential knowledge on the effectiveness of the MICP process for very coarse sands. Although the very coarse sand in this study had mean particle size of ${1820}\, {\upmu \hbox {m}}$, which is outside the optimum compatibility region for bio-cementation, it was successfully cemented with excellent uniformity when very low urease activity bacteria were used for both medium and high optical densities. MICP on very coarse-grained materials is successful when urease activities less than 10 mmol/L/h are used (pre-injection urease activity measurements at a bacterial $OD_{600}$ = 1 to urea (1.1 M) ratio 1:9) with bacterial populations with optical densities equal or higher than 2.

## Methods

### Preparation of bacteria with the desired urease activity

The bacterium *Sporosarcina pasteurii* was used as its urease-synthesis behaviour is well-defined^1^. In the study for preparation of bacteria with the desired urease activity, batch experiments were conducted under aerobic conditions and in a sterile environment. Bacteria were first extracted from the bacterial growth medium (nutrient broth) consisting of 20 g/L yeast extract, 10 g/L ammonium sulphate, 20 g/L agar, and 0.13 M Tris buffer (base). After 24 h of incubation at ${30}\, ^{\circ }\hbox {C}$, the culture was harvested and stored at ${4}\, ^{\circ }\hbox {C}$. The bacteria colonies were introduced into nutrient broth liquid-NBL (liquid nutrient medium without agar), which was then placed in a shaking incubator for 24 h and subsequently stored at ${4}\, ^{\circ }\hbox {C}$. The urease activity of these bacteria was monitored on a daily basis up until Day 42. Every 3–5 days 2 mL of bacterial suspension were introduced into 300 mL of new nutrient broth liquid and placed in incubation to reach an $OD_{600}$ of 1 ($4x10^8$ cells per mL). The urease activity of the new bacteria was also monitored on Day 1 and 3. On Day 3, 2 mL from the latter bacterial suspension were introduced to 300 mL of new nutrient broth and placed in incubation to reach an $OD_{600}$ of 1 ($4x10^8$ cells per mL) to grow a newer generation. The process was repeated in the same way until Day 42 and Generation VI. The bacterial suspensions were stored at all times at ${4}\, ^{\circ }\hbox {C}$.

The protocol followed to measure the urease activity required inoculation of urea solution of 18 mL with a concentration of 1.11 M with 2 mL of bacterial suspension at an $OD_{600}$ of 1. An electrical conductivity meter was used to record the change over a time window and the amount of hydrolysed urea was calculated using the equation^41^:

1 $$\begin{aligned} \text {Urea Hydrolysed (mM)}=\text {Conductivity (mS)} *11.11 \end{aligned}$$

### Quantitative Polymerase Chain Reaction (qPCR) analysis

The qPCR tests were conducted in triplicates and the steps followed are summarised below:

1. RNA extraction: 2 mL of culture per treatment were centrifuged and the supernatant was subsequently removed. ${100}\, {\upmu \hbox {L}}$ of RLT buffer containing ${10}\, {\upmu \hbox {L}}$ of 10 mg/mL lysozyme and ${20}\, {\upmu \hbox {L}}$ of 10 mg/mL proteinase K were added to the pellet and mixed by vortexing. Samples were incubated at room temperature for 10 min while mixing every 2 min. An extra ${350}\, {\upmu \hbox {L}}$ of buffer RLT were added in each sample and then vortexed vigorously. ${250}\, \upmu \hbox {L}$ of absolute ethanol were then added, samples were mixed by vortexing and finally transferred to RNeasy spin columns. The columns were centrifuged for 30 sec at 8000xg and the flow through was discarded. ${700}\, {\upmu \hbox {L}}$ of RW1 buffer were added and the samples were centrifuged for another minute. The flow through was discarded. Two wash steps were performed within the spin columns by adding ${500}\, {\upmu \hbox {L}}$ of RPE buffer and by centrifuging samples for 1 min at 8000xg. The flow though was discarded and the columns were placed into fresh 2 mL collection tubes following centrifuging for 3 min at 8000xg to dry the columns. ${30}\, {\upmu \hbox {L}}$ of RNase-free water were added in the columns which were centrifuged for an extra minute at the same speed to elute the RNA.
2. RNA integrity, purity and concentration: RNA concentrations were measured using a Nanodrop spectrophotometer (Thermo Scientific). RNA integrity was assessed using the Agilent Bioanalyzer. RNA purity and concentration were measured using a NanoDrop spectrophotometer. The ratio of the readings at 260 nm and 280 nm (A260/A280) provides an estimate of the purity of RNA with respect to contaminants that absorb in this range of the spectrum. This ratio was larger than 2.0 for all samples indicating good purity. The absorbance peak was at 260 nm for all samples, which is the correct peak for nucleic acids.
3. Reverse transcription: 500 ng RNA were reverse transcribed using the Qiagen Quantitect reverse transcription in a 10 $\upmu$L reaction according to the manufacturer’s instructions. The RT kit protocol involves a mandatory gDNA wipeout step for 4min at ${42}^{\circ }\hbox {C}$ for the samples. RT duplicate reactions were performed for one of the samples. 3 $\upmu$L of RT mastermix containing (per reaction), 0.5 $\upmu$L of OligdT and random primer mix, 0.5 $\upmu$L of reverse transcriptase and 2 $\upmu$L RT buffer were added to 7 $\upmu$L of RNA mixed with gDNA wipeout buffer and the samples were incubated at ${42}^{\circ }\hbox {C}$ for 20 min followed by ${95}^{\circ }\hbox {C}$ for 5 min. The completed reaction was diluted 10-fold with 0.5 $\upmu$g/mL tRNA in nuclease-free water.
4. Quantitative real-time PCR (qPCR)
  **(a) Assay validation**
  All qPCR reactions were pipetted robotically using a Qiagility workstation (Qiagen). A pool of all six cDNAs was prepared and 4 $\upmu$L amplified for each of the four primer sets in a 20 $\upmu$L reaction using Qiagen Quantifast SYBR Green mix with each primer at a final concentration of 500 nmol/L.
  The Master mix per reaction included ${10}\,{\upmu \hbox {L}}$ of Quantifast SYBR Green mix, ${1}\, {\upmu \hbox {L}}$ of the forward primer at ${10}\, \upmu \hbox {mol/L}$, ${1}\, {\upmu \hbox {L}}$ of the reverse primer at ${10}\, \upmu \hbox {mol/L}$, ${4}\, {\upmu \hbox {L}}$ of water and the template of pooled cDNA at ${4}\, {\upmu \hbox {L}}$. Amplification parameters: ${95}^{\circ }\hbox {C}$ for 5 min followed by 40 cycles of ${95}^{\circ }\hbox {C}$ for 10 sec, ${60}^{\circ }\hbox {C}$ for 20 sec using a Rotor-Gene Q qPCR machine. The Melt curves were derived by ramping from ${65}^{\circ }\hbox {C}$ to ${95}^{\circ }\hbox {C}$ rising by ${1}^{\circ }\hbox {C}$ per step. Melt curves were checked for product specificity (single peak) and for the presence of primer dimers.
  The PCR products went through electrophoresis in order to check that a single product of the expected size amplified for each assay.The amplified samples were run on an agarose gel (2% size-select E-gel Invitrogen) together with a 50 bp ladder marker (New England Biolabs) . The product for each assay purified using an equal volume of microClean. 10 $\upmu$L of TE buffer were added to each tube and the DNA was quantified on a Nanodrop 2000 spectrophotometer. The concentration was converted to copies per $\upmu$L using Avogadro’s constant and the expected extinction coefficient for each amplicon to generate a standard of known copy number. This was diluted to 5x106 copies/μL with tRNA (0.5 $\upmu$g/mL) and a 10-fold serial dilution to 5x100 copies/$\upmu$L prepared. Two microlitres of each dilution were used in the standard reactions of known copy number. The Master mix per reaction included ${5}\, {\upmu \hbox {L}}$ of Quantifast SYBR Green mix, ${0.5}\, {\upmu \hbox {L}}$ of the forward primer at ${10}\, \upmu \hbox {mol/L}$, ${0.5}\, {\upmu \hbox {L}}$ of the reverse primer at ${10}\, \upmu \hbox {mol/L}$, ${2}\, {\upmu \hbox {L}}$ of water and the standard template at ${2}\, {\upmu \hbox {L}}$.
  The amplification parameters were as above. All assays were efficient ($\ge$94%) and linear from 107 to 101 copies per reaction.
  **(b) qPCR of samples and standards**
  Two microlitres of cDNA were amplified in a 10 $\upmu$L reaction using the reactions specified below. The no-template control reaction contained 2 $\upmu$L of $\upmu$g/mL). DNA standards (107-101 copies/rxn) for each gene were included in each run. The Master mix per reaction included ${5}\, {\upmu \hbox {L}}$ of Quantifast SYBR Green mix, ${0.5}\, {\upmu \hbox {L}}$ of the forward primer at ${10}\, \upmu \hbox {mol/L}$, ${0.5}\, {\upmu \hbox {L}}$ of the reverse primer at ${10}\, \upmu \hbox {mol/L}$, ${2}\, {\upmu \hbox {L}}$ of water and the templates at ${2}\, {\upmu \hbox {L}}$ (standard, tRNA or cDNA depending on the reaction).
  The forward and reverse primers designed to amplify the Ure-C gene are 1-GTGTACCTGCTAAACTTGGCTTG-23 and 1-TCCGTTGTCACGTCATTCCA-20, respectively.
5. Data normalisation and statistical analysis: Data collection was performed at the end of each annealing/extension step. Copy numbers/reaction were derived from the standard curves using the Rotor-Gene Q software. Three reference genes were used to calculate the normalisation factor. The forward and reverse primers, all at 500 nmol/L are listed below:
  atpD gene: 1-TCCGCTGGTACGTAGATTGC-20 and 1-ATGCCTTCAGCGGTTGGTTA-20
  gyrA gene: 1-TGGCCGTGGAATTCAAGGAA-20 and 1-AGCCTTTTGCCGTTCGACTA-20
  rpoD gene: 1-CGTCGCCTTCAATGATTCGC-20 and 1-TAATGACCCGGTTCGCATGT-20
  When the normalisation procedure is appropriate, the normalised RT duplicates should be similar in copy number as they originate from the same source of RNA. The normalisation factor for each sample was used to normalise ureC copy number. The melt curves exhibited a single peak at the expected temperature.

### Preparation of flask and sand columns experiments

For the study of the role of urease activity on the bio-cementation of coarse sand specimens, bacterial populations were cultivated in a similar manner. The bacterial growth medium (nutrient broth) consisted of 20 g/L yeast extract, 10 g/L ammonium sulphate, 20 g/L agar, and 0.13 M Tris buffer (base). After 24 h of incubation at ${30}\, ^{\circ }\hbox {C}$, the culture was harvested and stored at ${4}\, ^{\circ }\hbox {C}$. The bacteria colonies were introduced into nutrient broth liquid-NBL (liquid nutrient medium without agar), which was then placed in a shaking incubator for 24 h and subsequently stored at ${4}\, ^{\circ }\hbox {C}$. The cementation solution used in this study comprised 0.375 M urea, 0.25 M calcium chloride ($CaCl_2$), and 3g/L nutrient broth. This recipe is consistent with several previous studies that showed effective MICP treatment^2^.

For the preliminary (flask) experiments, three different urease activities were used at three different bacterial densities ($OD_{600}$ of 1, 2 and 3) to cement samples in triplicates. For the sand column experiments, five different urease activities were used at two different bacterial densities ($OD_{600}$ of 2 and 3, -$8\times 10^8$ and $12\times 10^8$ cells per mL-, respectively) to cement samples in duplicates.

Urease activity was measured following two protocols: (1) a standard protocol by Whiffin^1^ in which measurements of the activity are taken from bacterial suspension at optical densities of 1.0 and (2) a protocol which closely describes the reactions within the flasks and sand columns. The standard protocol required dilution of the bacterial suspensions to reach an optical density of $OD_{600}=1$ ($8x10^8$ cells per mL). A urea solution of 18 mL with a concentration of 1.11 M was inoculated with 2 mL of bacteria 1:9^41^. An electrical conductivity meter was used to record the change over a hydrolysed urea was calculated based on the protocol by Whiffin^41^ which was described previously. The urease activity measured following the standard protocol at $OD_{600}=1$ fell in a range between 0.5 and 290 mmol/L/h, which is quite large and spans the ranges of activity reported in literature. The second protocol targeted the measurement of the reaction rates within experiments. A urea solution of 10 mL with a concentration of 0.75 M was inoculated with 10 mL of bacterial suspension (0.375 M in the total volume of bacteria and urea mixture). The volume ratio of bacterial suspension and urea was 1:1. Tables S1 and S2 present the values of urease activity and specific urease activity of all bacterial cultures used in this study. These measurements were taken before injecting the bacteria to ensure that good testing conditions had been achieved.

The bacteria solution was only injected once at the beginning of the process for both types of experiments. The retention time between two subsequent injections was 24 hours for both bacteria and cementation injections. The number of injections of the cementation solution was fixed at 15 in order to isolate the effects of the bacterial parameters.

For the flask experiments no effluent was allowed, as without the presence of a granular medium, bacteria would be completely flushed out. Once the injection phase was completed, the culture liquid in each of the flask experiments was filtered with a filter paper. Both the filtered paper containing residues and the conical flask were dried at ${100}\, ^{\circ }\hbox {C}$ for 24 h.

For the sand column experiments, injections were made from the top of the specimens via gravity, as this method has generally been shown to result in uniform samples^40^. Before each new chemical solution injection the excess solution was allowed to freely drain out, but outflow was prevented when new liquid was introduced. In all phases the sample was flooded. At the end of the last retention period, specimens were extracted from the moulds with care to minimise disturbance and were dried at ${100}\, ^{\circ }\hbox {C}$ for 24h. The ends of the specimens were then trimmed to eliminate potentially disturbed or uneven zones. The specimens were 70mm in diameter and 150mm in height. The mean particle size of the subrounded silica sand was ${1820}\, {\upmu \hbox {m}}$ with a $D_{10}$ equal to ${1350}\, \, {\upmu \hbox {m}}$ and a $D_{90}$ equal to ${2100}\, {\upmu \hbox {m}}$.

The specimens generated in this study were used as a proxy to natural sandstone for further laboratory testing (determination of engineering properties like unconfined compressive strength, tensile strength, point load index etc.). In these types of testing, any presence of moisture would had given false measurements. To avoid such problems it is common practice to oven dry the MICP-treated soil samples^24,29,31,35,38,44^. However, incubating the calcium carbonate likely results to conversion of the metastable minerals to calcite and/or could result in recrystallization of calcite. Therefore, the results of this study are limited to lab-based applications.

### Carbonate content

The precipitated calcium carbonate in the flask experiments was measured as the difference between the masses of the dried flask and filter paper containing the precipitates ($W_1$) and the masses of the dried flask and filter paper after introducing hydrochloric acid ($W_2$) adjusted to the stoichiometry of the following reaction:

$$\begin{aligned} CaCO_{3(s)} + 2HCl_{(aq)} -> CaCl_{2(aq)} + CO_{2(g)} + H_{2}O_{(l)} \end{aligned}$$

which yields:

2 $$\begin{aligned} m_{CaCO_{3}}=100 \frac{W_2-W_1}{111-100} \end{aligned}$$

The uniformity of the sand specimens was assessed by measuring the carbonate content at various points in the sample according to the procedure defined by the ASTM standard method^51^. A 30g dried and ground sample was treated with 30 mL of hydrochloric acid (*HCl*) 2.5 mol. Calcite ($CaCO_{3}$) is dissolved according to the reaction stated previously.

Carbon dioxide is released into a chamber equipped with a pressure gauge. The pressure reading is translated into amount of carbon dioxide and with the aid of the stoichiomentry of the above reaction the amount of calcium carbonate is calculated. The degree of cementation is expressed in this study as weight of calcite over the total weight of the sample tested (in percent).

The cementation level of each specimen was measured at 5 points across the height of the sample and profiles were constructed as shown in Fig. 4. These profiles were then used to derive the average cementation and the variance, which is the metric defined for assessing the uniformity of the samples.

### Micro-CT scanning and SEM imaging

Scans of the bio-treated samples were performed with the X-ray $\mu$-CT high energy micro-tomography scanner (X-Tec Systems) with the following settings: 160 kV, 110 uA with a use of a 0.5 mm copper filter. Four specimens (samples generated with bacteria of the highest and lowest urease activities at both optical densities under investigation) were scanned with a voxel size of the images of around 30 $\upmu$m. The inner structure, mineralogy and final porosity of the samples were investigated based on the results of the microCT analysis. Stacks of images were obtained and transferred to the ImageJ software^52,53^. The images were then binarised and were analysed via the BoneJ plugin^54^ to obtain the porosity and distribution of voids^55^. More information on the steps how the plugin was used is presented in the supplementary material.

SEM images of bio-treated samples were taken to investigate the carbonate crystals and the bonding network. The microscopy investigation was carried out with a Hitachi S-5500 scanning electron microscope. All bio-treated samples were placed in the oven and dried at ${100}\, ^{\circ }\hbox {C}$ for 24h before conducting the microscopy analysis.

### Monitoring of the process

The MICP treatment of the very coarse particles was closely monitored during the injection phase through measurements of pH, urease activity and optical density in the effluent, while the flow rates during each injection were also recorded. Optical density of the effluent was recorded for every 75 mL to 100 mL of the effluent during the first injection process giving a total of 3-4 measurements, as the total injected volume was about 300 mL. The pH values were measured in similar subsets of the effluent for all injections. The purpose for these measurements was to introduce control parameters for the prediction of the success of the MICP treatment during the injection phase.

## Supplementary information

Supplementary Information

## Data Availability

The data that support the findings of this study are available from the corresponding authors upon reasonable request.

## References

[1] Whiffin, V. S. *Microbial CaCO3 Precipitation for the Production of Biocement*. Ph.D. thesis, Murdor University (2004).

[2] DeJong JT, Fritzges MB, Nüsslein K (2006). Microbially induced cementation to control sand response to undrained shear. J. Geotech. Geoenviron. Eng..

[3] DeJong JT, Mortensen BM, Martinez BC, Nelson DC (2010). Bio-mediated soil improvement. Ecol. Eng..

[4] Dejong JT (2013). Biogeochemical processes and geotechnical applications: progress, opportunities and challenges. Geotechnique.

[5] Montoya BM, DeJong JT, Boulanger RW (2013). Dynamic response of liquefiable sand improved by microbial-induced calcite precipitation. Geotechnique.

[6] Montoya BM, DeJong JT (2015). Stress–strain behavior of sands cemented by microbially induced calcite precipitation. J. Geotech. Geoenviron. Eng..

[7] Jiang N-J, Soga K, Kuo M (2017). Microbially induced carbonate precipitation for seepage-induced internal erosion control in sand-clay mixtures. J. Geotech. Geoenviron. Eng..

[8] Cheng, L., Shahin, M. & Cord-Ruwisch, R. Soil stabilisation by microbial-induced calcite precipitation (MICP): investigation into some physical and environmental aspects. *7th International Congress on Environmental Geotechnics* **64**, 1105–1112 (2014).

[9] Umar, M., Kassim, K. A. & Ping Chiet, K. T. Biological process of soil improvement in civil engineering: a review. *J. Rock Mech. Geotech. Eng.* **8**, 767–774. 10.1016/j.jrmge.2016.02.004(2016).

[10] Bella G, Barbero M, Barpi F, Borri-Brunetto M, Peila D (2017). An innovative bio-engineering retaining structure for supporting unstable soil. J. Rock Mech. Geotech. Eng..

[11] Konstantinou, C. & Biscontin, G. Soil enhancement via microbially induced calcite precipitation. In *Proceedings of the 10th International Symposium on Geotechnical Aspects of Underground Construction in Soft Ground*, Taylor & Francis (2021).

[12] Torres-Aravena ÁE (2018). Can microbially induced calcite precipitation (MICP) through a ureolytic pathway be successfully applied for removing heavy metals from wastewaters?. Crystals.

[13] Li M, Cheng X, Guo H (2013). Heavy metal removal by biomineralization of urease producing bacteria isolated from soil. Int. Biodeterior. Biodegrad..

[14] Castro-Alonso MJ (2019). Microbially induced calcium carbonate precipitation (MICP) and its potential in bioconcrete: microbiological and molecular concepts. Front. Mater..

[15] Harbottle, M. J., Botusharova, S. P. & Gardner, D. R. Self-healing soil: Biomimetic engineering of geotechnical structures to respond to damage. In *Proceedings of the 7th International Congress on Environmental Geotechnics*, 1121–1128 (Melbourne, 2014).

[16] Erşan YÇ, Hernandez-Sanabria E, Boon N, De Belie N (2016). Enhanced crack closure performance of microbial mortar through nitrate reduction. Cem. Concr. Compos..

[17] Konstantinou, C., Biscontin, G., Jiang, N.-J. & Soga, K. Application of microbially induced carbonate precipitation (MICP) to form bio-cemented artificial sandstone. *J. Rock Mech. Geotech. Eng.* **13**. 10.1016/j.jrmge.2021.01.010 (2021).

[18] De Muynck W, De Belie N, Verstraete W (2010). Microbial carbonate precipitation in construction materials: a review. Ecol. Eng..

[19] Crittenden, J. C., Trussell, R. R., Hand, D. W., Howe, K. J. & Tchobanoglous, G. *Water Treatment principle and design* (Wiley, 2012), 3rd edn.

[20] Stocks-Fischer S, Galinat JK, Bang SS (1999). Microbiological precipitation of CaCO3. Soil Biol. Biochem..

[21] Wang Y, Soga K, Dejong JT, Kabla AJ (2019). Microscale visualization of microbial-induced calcium carbonate precipitation processes. J. Geotech. Geoenviron. Eng..

[22] Cheng L, Shahin MA, Chu J (2019). Soil bio-cementation using a new one-phase low-pH injection method. Acta Geotech..

[23] Matsubara H, Yamada T (2020). Mathematical modelling and simulation of microbial carbonate precipitation: the urea hydrolysis reaction. Acta Geotech..

[24] van Paassen, L. *Biogrout: Ground Improvement by Microbially Induced Carbonate Precipitation*. Phd thesis, Delft University of Technology (2009).

[25] Al Qabany A, Soga K, Santamarina C (2012). Factors affecting efficiency of microbially induced calcite precipitation. J. Geotech. Geoenviron. Eng..

[26] Martinez BC (2013). Experimental optimization of microbial-induced carbonate precipitation for soil improvement. J. Geotech. Geoenviron. Eng..

[27] Cheng L, Cord-Ruwisch R, Shahin MA (2013). Cementation of sand soil by microbially induced calcite precipitation at various degrees of saturation. Can. Geotech. J..

[28] Dadda A (2019). Characterization of contact properties in biocemented sand using 3D X-ray micro-tomography. Acta Geotech..

[29] Terzis D, Laloui L (2018). 3-D micro-architecture and mechanical response of soil cemented via microbial-induced calcite precipitation. Sci. Rep..

[30] Mahawish A, Bouazza A, Gates WP (2018). Effect of particle size distribution on the bio-cementation of coarse aggregates. Acta Geotech..

[31] Al Qabany A, Soga K (2013). Effect of chemical treatment used in MICP on engineering properties of cemented soils. Geotechnique.

[32] Zhao Q (2014). Factors affecting improvement of engineering properties of MICP-treated soil catalyzed by bacteria and urease. J. Mater. Civ. Eng..

[33] Dawoud, O. *The Applicability of Microbially Induced Calcite Precipitation (MICP) for Soil Treatment*. Ph.D. thesis, University of Cambridge (2015).

[34] Feng K, Montoya BM (2015). Influence of confinement and cementation level on the behavior of microbial-induced calcite precipitated Sands under monotonic drained loading. J. Geotech. Geoenviron. Eng..

[35] Cheng L, Shahin MA, Mujah D (2017). Influence of key environmental conditions on microbially induced cementation for soil stabilization. J. Geotech. Geoenviron. Eng..

[36] Cui MJ, Zheng JJ, Zhang RJ, Lai HJ, Zhang J (2017). Influence of cementation level on the strength behaviour of bio-cemented sand. Acta Geotech..

[37] Mitchell JK, Santamarina JC (2005). Biological considerations in geotechnical engineering. J. Geotech. Geoenviron. Eng..

[38] Mahawish A, Bouazza A, Gates WP (2018). Improvement of coarse sand engineering properties by microbially induced calcite precipitation. Geomicrobiol. J..

[39] Cheng L, Cord-Ruwisch R (2014). Upscaling effects of soil improvement by microbially induced calcite precipitation by surface percolation. Geomicrobiol. J..

[40] Mujah D, Shahin MA, Cheng L (2017). State-of-the-art review of biocementation by microbially induced calcite precipitation (MICP) for soil stabilization. Geomicrobiol. J..

[41] Whiffin VS, van Paassen LA, Harkes MP (2007). Microbial carbonate precipitation as a soil improvement technique. Geomicrobiol. J..

[42] Wang Y, Soga K, Dejong JT, Kabla AJ (2019). A microfluidic chip and its use in characterising the particle-scale behaviour of microbial-induced calcium carbonate precipitation (MICP). Geotechnique.

[43] Wang, Y., Konstantinou, C., Soga, K., Dejong, J. T. & Kabla, A. J. Enhancing strength of MICP-treated sandy soils: from micro to macro scale. *arXiv preprint* 1–23, 2006.15760 (2020).

[44] Lin H, Suleiman MT, Brown DG, Kavazanjian E (2015). Mechanical behavior of sands treated by microbially induced carbonate precipitation. J. Geotech. Geoenviron. Eng..

[45] Ma L, Pang AP, Luo Y, Lu X, Lin F (2020). Beneficial factors for biomineralization by ureolytic bacterium Sporosarcina pasteurii. Microbial Cell Factories.

[46] Benini, S., Rypniewski, W. R., Wilson, K. S., Ciurli, S. & Mangani, S. The complex of Bacillus pasteurii urease with $$\beta$$-mercaptoethanol from X- ray data at 1.65-Å resolution. *J. Biol. Inorganic Chem.* **3**, 268–273. 10.1007/s007750050231 (1998). 10.1007/s007750050014 10766443

[47] Kappaun K, Piovesan AR, Carlini CR, Ligabue-Braun R (2018). Ureases: historical aspects, catalytic, and non-catalytic properties - A review. J. Adv. Res..

[48] Benini S, Cianci M, Mazzei L, Ciurli S (2014). Fluoride inhibition of Sporosarcina pasteurii urease: structure and thermodynamics. J. Biol. Inorg. Chem..

[49] Wang, Y., Soga, K., Dejong, J. T. & Kabla, A. J. Effects of bacterial density on growth rate and characteristics of microbial-induced CaCO3 precipitates: a particle-scale experimental study. *Geotech. Geoenviron. Eng, ASCE J*. 10.1016/(ASCE)GT.1943-5606.0002509 (2021).

[50] Wang, Y. *Microbial-Induced Calcium Carbonate Precipitation: from micro to macro scale*. Ph.D. thesis, University of Cambridge (2018).

[51] ASTM. Standard Test Method for Rapid Determination of Carbonate Content of Soils. *ASTM International* 1–5, 10.1520/D4373-14. (2014).

[52] Rueden CT (2017). Image J2: ImageJ for the next generation of scientific image data. BMC Bioinform..

[53] Schindelin J (2012). Fiji: an open-source platform for biological-image analysis. Nat. Methods.

[54] Doube M (2010). BoneJ: free and extensible bone image analysis in ImageJ. Bone.

[55] Dougherty R, Kunzelmann K-H (2007). Computing local thickness of 3D structures with ImageJ. Microsc. Microanal..

