# Supplementary material for: The role of bacterial urease activity on the uniformity of carbonate precipitation profiles of bio-treated coarse sand specimens
Source: Sci Rep. 2021 Mar 17;11:6161. doi: 10.1038/s41598-021-85712-6 (PMC7969948; doi:10.1038/s41598-021-85712-6)
Supplement: Supplementary file 1 — Supplementary Information [file 41598_2021_85712_MOESM1_ESM.pdf]

The role of bacterial urease activity on the uniformity of carbonate precipitation profiles of bio-treated coarse sand specimens

Charalampos Konstantinou[1]*, Yuze Wang[2]*, Giovanna Biscontin[3], and Kenichi Soga[4]

Author 1

- Charalampos Konstantinou ***(corresponding author)***
- Department of Engineering, University of Cambridge, UK. Email: ck494@cam.ac.uk ORCID: https://orcid.org/0000-0002-4662-5327

Author 2

- Yuze Wang, PhD ***(corresponding author)***
- Department of Ocean Science and Engineering, Southern Marine Science and Engineering Guangdong Laboratory (Guangzhou), Southern University of Science and Technology，China. E-mail: wangyz@sustech.edu.cn, ORCID: https://orcid.org/0000-0003-3085-5299.

Author 3

- Giovanna Biscontin, PhD
- Department of Engineering, University of Cambridge, UK. Email: gb479@cam.ac.uk ORCID: https://orcid.org/0000-0002-4662-5650

Author 4

- Kenichi Soga, PhD, FREng, FICE
- Department of Civil and Environmental Engineering, University of California, Berkeley, CA 94720, USA. Email: soga@berkeley.edu

Supplementary Material: This document includes supplementary figures, tables and discussion

Table S1. The urease activities used in this study measured following the protocol suggested by Whiffin (2004).

	**Urease activity (mmol/L/h)**	**Specific urease activity (mmol/L/h/OD)**	**Specific urease activity (mmol/L/h/cell)**
**Flask experiments**			
High	294.9	294.9	$3.686 * 10^{-7}$
Medium	66.1	66.1	$8.268 * 10^{-8}$
Low	9.8	9.8	$1.226 * 10^{-8}$
**Sand columns experiments**			
Urease Activity 1	237.4	237.4	$2.969 * 10^{-7}$
Urease Activity 2	112.9	112.9	$1.412 * 10^{-7}$
Urease Activity 3	44.1	44.1	$5.516 * 10^{-8}$
Urease Activity 4	7.5	7.5	$9.375 * 10^{-9}$
Urease Activity 5	0.45	0.45	$5.625 * 10^{-10}$

Table S2. The urease activities used in this study measured following a protocol that best simulates the conditions within the actual experiments

	**Urease activity (mmol/L/h)**			**Specific urease activity (mmol/L/h/OD)**			**Specific urease activity (mmol/L/h/cell)**		
	**OD_{600}=1**	**OD_{600}=2**	**OD_{600}=3**	**OD_{600}=1**	**OD_{600}=2**	**OD_{600}=3**	**OD_{600}=1**	**OD_{600}=2**	**OD_{600}=3**
**Flask experiments**									
High	149.9	231.9	355.5	149.9	115.9	118.5	$3.748 * 10^{-8}$	$2.898 * 10^{-8}$	$2.963 * 10^{-8}$
Medium	26.5	38.2	46.1	26.5	19.1	15.4	$6.625 * 10^{-9}$	$4.775 * 10^{-9}$	$3.85 * 10^{-9}$
Low	2.8	4.7	6.9	2.8	2.4	2.3	$7 * 10^{-10}$	$6 * 10^{-10}$	$5.75 * 10^{-10}$
**Sand columns experiments**									
Urease activity 1		266.6	344.5		133.3	114.8		$3.333 * 10^{-8}$	$2.87 * 10^{-8}$
Urease activity 2		195.1	167.8		97.5	55.9		$2.438 * 10^{-8}$	$1.398 * 10^{-8}$
Urease activity 3		8.2	10.4		4.1	3.5		$1.025 * 10^{-9}$	$8.75 * 10^{-10}$
Urease activity 4		4.1	3.7		2.0	1.2		$5 * 10^{-10}$	$3 * 10^{-10}$
Urease activity 5		2.5	3.4		1.3	1.1		$3.25 * 10^{-10}$	$2.75 * 10^{-10}$

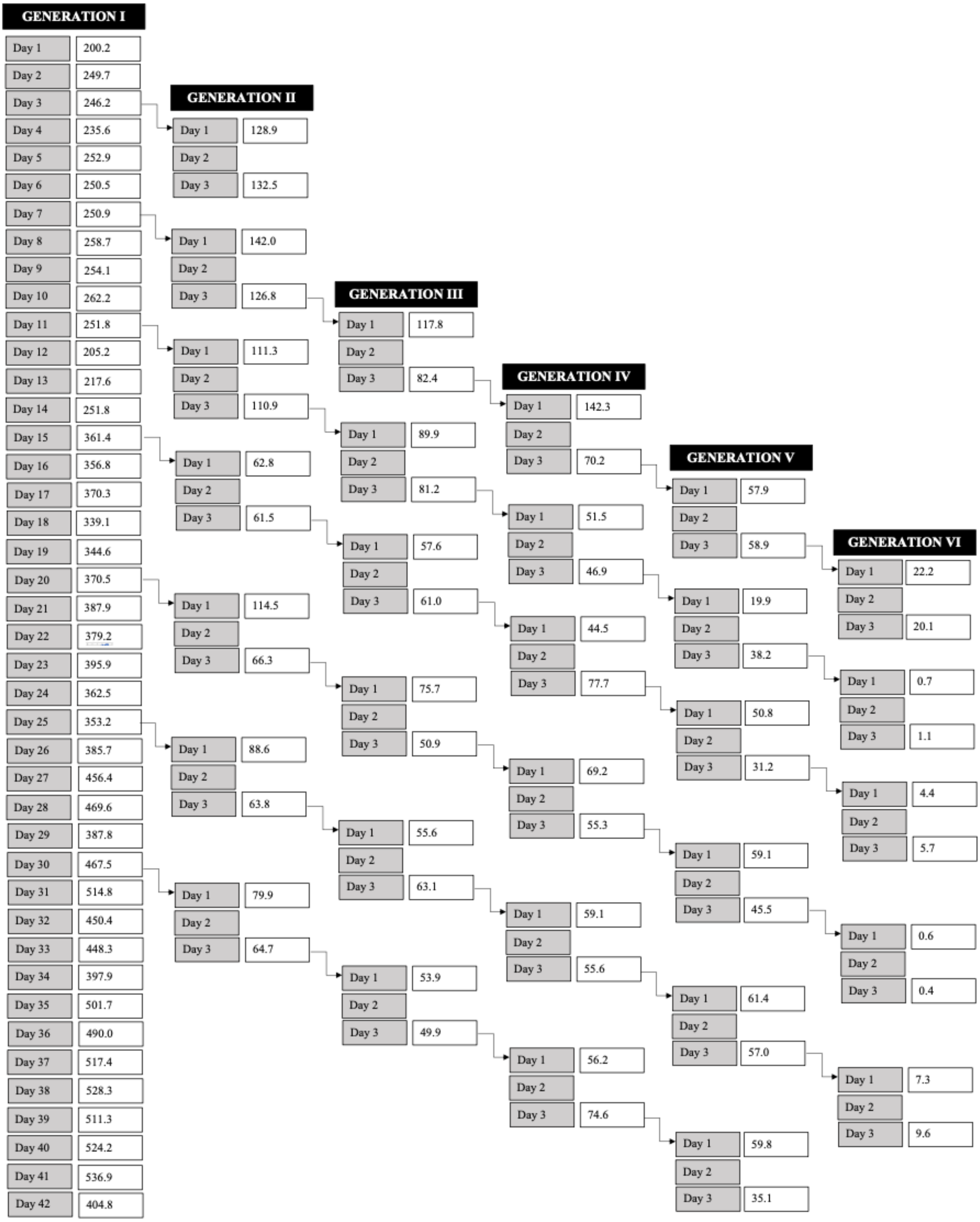

Figure S1. A procedure for delivering bacteria of a desired urease activity. The urease activity is measured in mmol/L/h. Bacteria were first extracted from the petri dish and introduced to the nutrient broth liquid (NBL). These bacteria are referred to as 'Generation I' and the urease activity was monitored on a daily basis up until Day 42. Every 3-5 days 2 mL of bacterial suspension were introduced into 300 mL of new nutrient broth liquid and placed in incubation to reach an OD_{600} of 1. The urease activity of the new bacteria (Generation II) was also monitored on Day 1 and 3. On Day 3, 2 mL from the Generation II bacteria were introduced to 300 mL of new nutrient broth and placed in incubation to reach an OD_{600} of 1 to grow Generation III. The process was repeated in the same way until Day 42 and Generation VI.

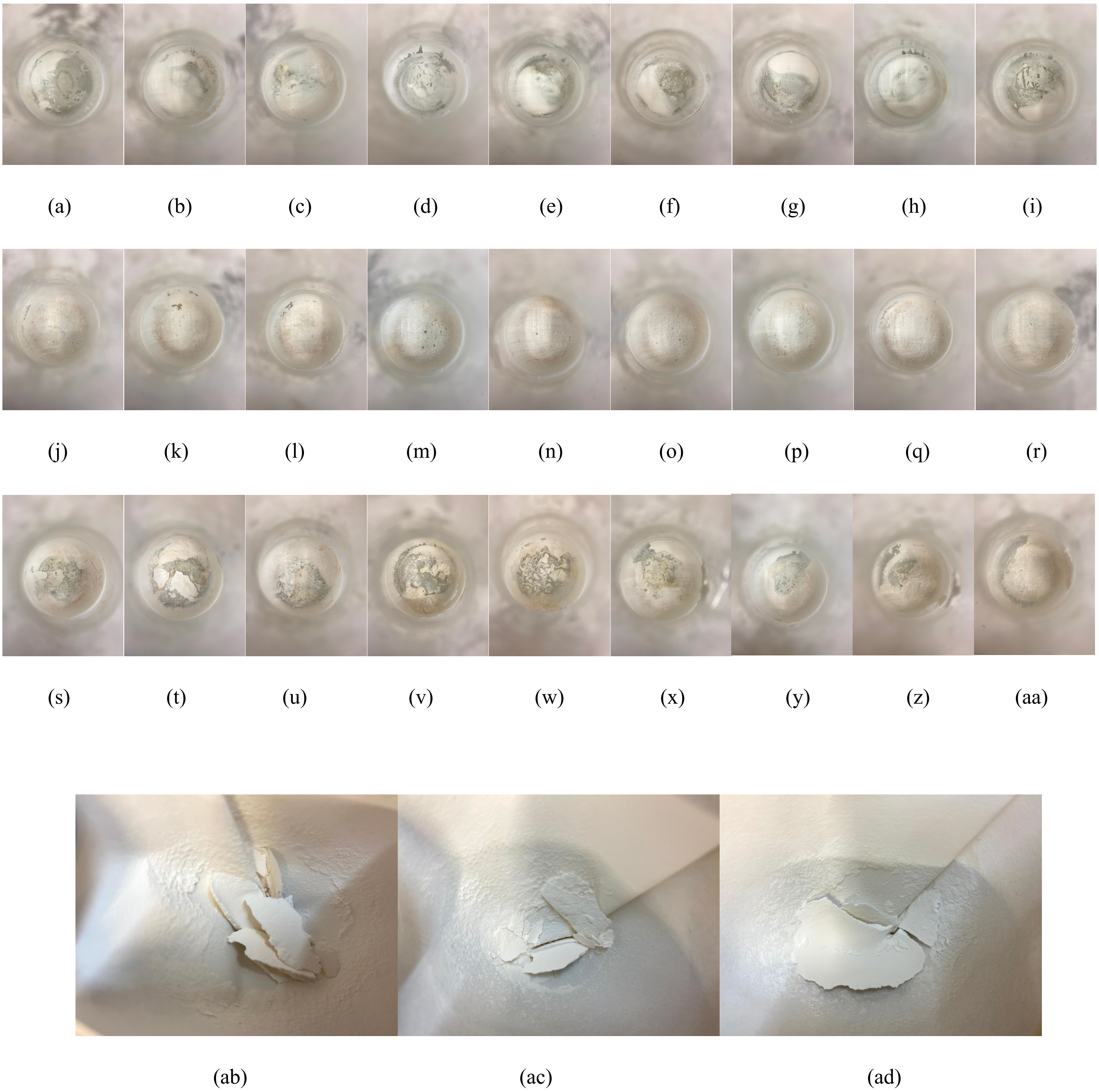

Figure S2. (a-aa) Precipitated calcium carbonate at the bottom of the flask experiments. (a-c) OD_{600} =1, urease activity at 294.9 mmol/L/h, (d-f) OD_{600} =2, urease activity at 294.9 mmol/L/h, (g-i) OD_{600} =3, urease activity at 294.9 mmol/L/h (j-l) OD_{600} =1, urease activity at 66.1 mmol/L/h, (m-o) OD_{600} =2, urease activity at 66.1 mmol/L/h, (p-r) OD_{600} =3, urease activity at 66.1 mmol/L/h, (s-u) OD_{600} =1, urease activity at 9.8 mmol/L/h, (v-x) OD_{600} =2, urease activity at 9.8 mmol/L/h and, (y-aa) OD_{600} =3, urease activity at 9.8 mmol/L/h. (ab-ad) Examples of precipitates retained on the filter paper during filtration.

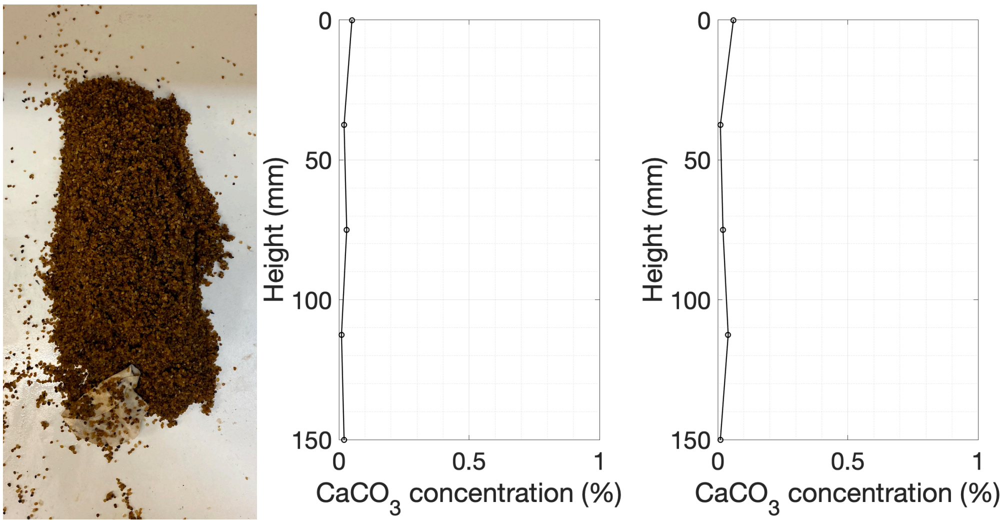

(a) (b). (c)

Figure S3. Blank column experiment without bacterial solution injection and fifteen injections of the cementation solution: (a) photograph of the sample taken after de-molding. (b-c) the two calcium carbonate concentration profiles.

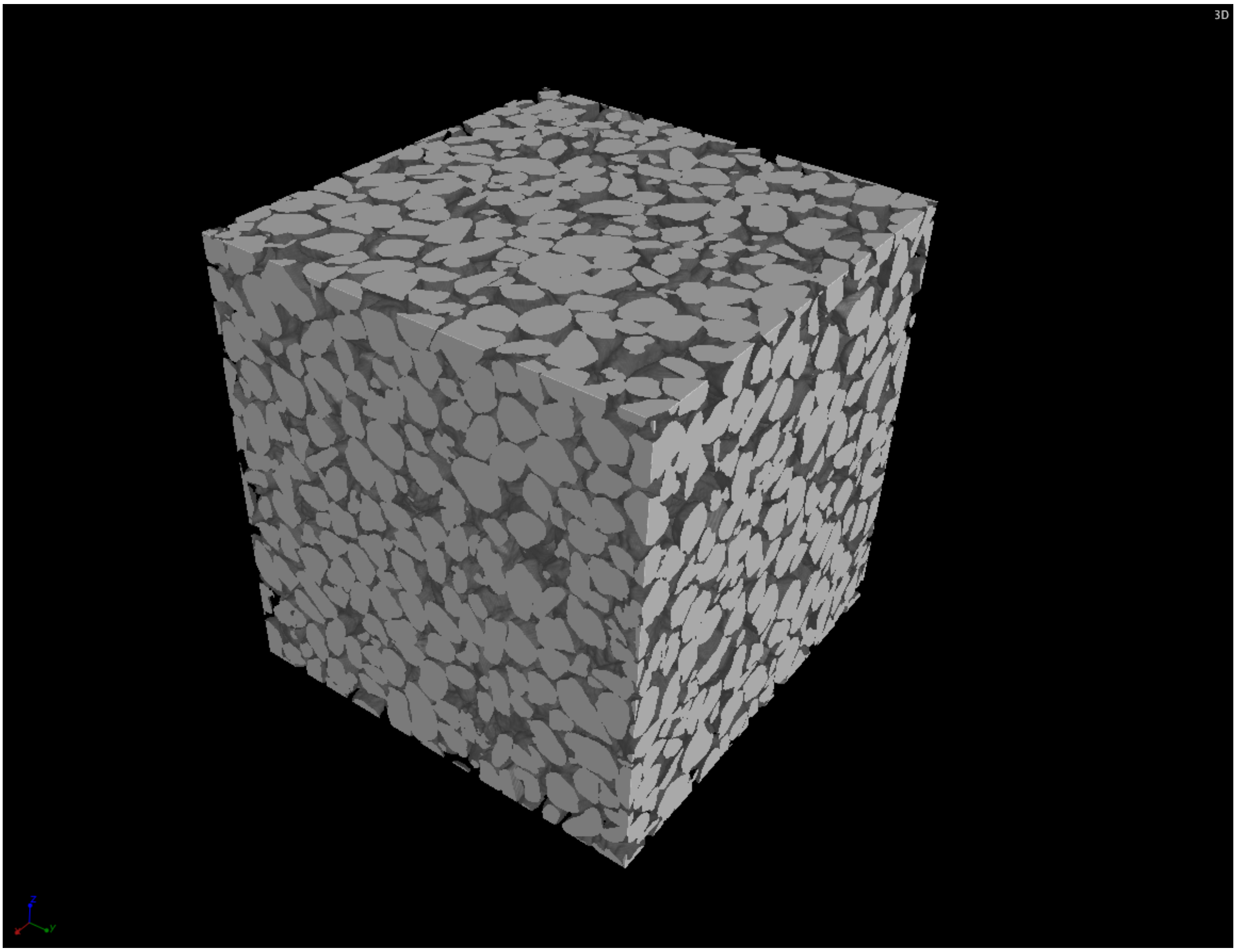

(a)

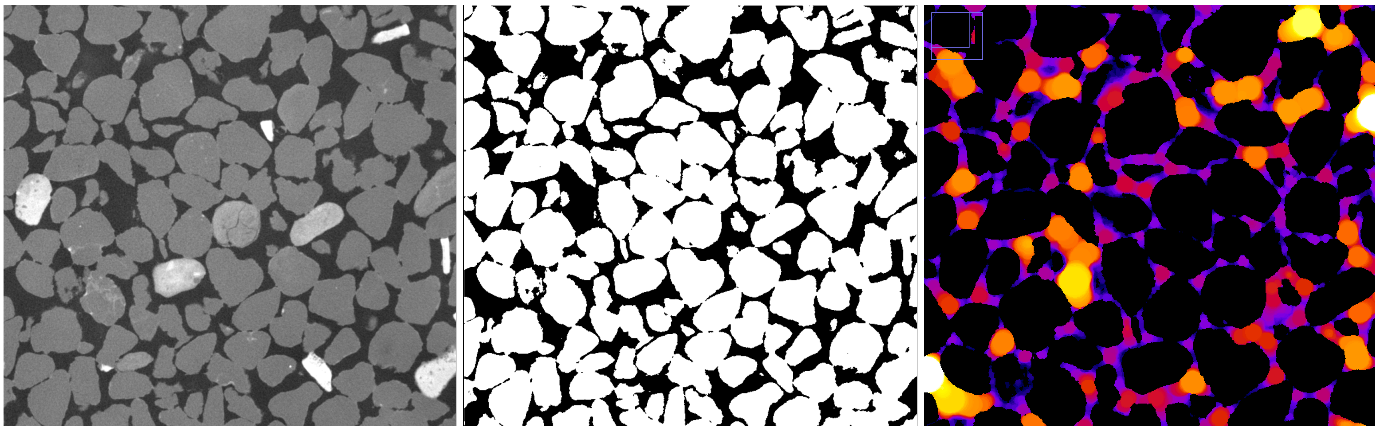

(b) (c) (d)

Figure S4. The calculation of the pore space histograms: (a) An example of MicroCT reconstruction of a cubic specimen (b) Slicing of the 3D reconstruction (c) Binarising of the slices (d) Calculating the pore space via the BoneJ plug-in. Stacks of images were obtained and transferred to the ImageJ software[52, 53]. The images were then binarised and were analysed via the BoneJ plugin[54] to obtain the porosity and distribution of voids[55].

The porosity is calculated based on the volume fraction function of BoneJ. The function calculates the fraction of a bone in a 3D image relative the whole image by counting the voxels representing the bone over the total number of voxel the image contains. In this case, the 'bone' is defined as grains and carbonate cement.

The pore space size histograms are calculated utilising the local thickness plugin by BoneJ. The thickness at a point (trabeculae for bones, pore space in this specific application) is defined as the diameter of the largest sphere that fits within the available structure whilst also containing the point.

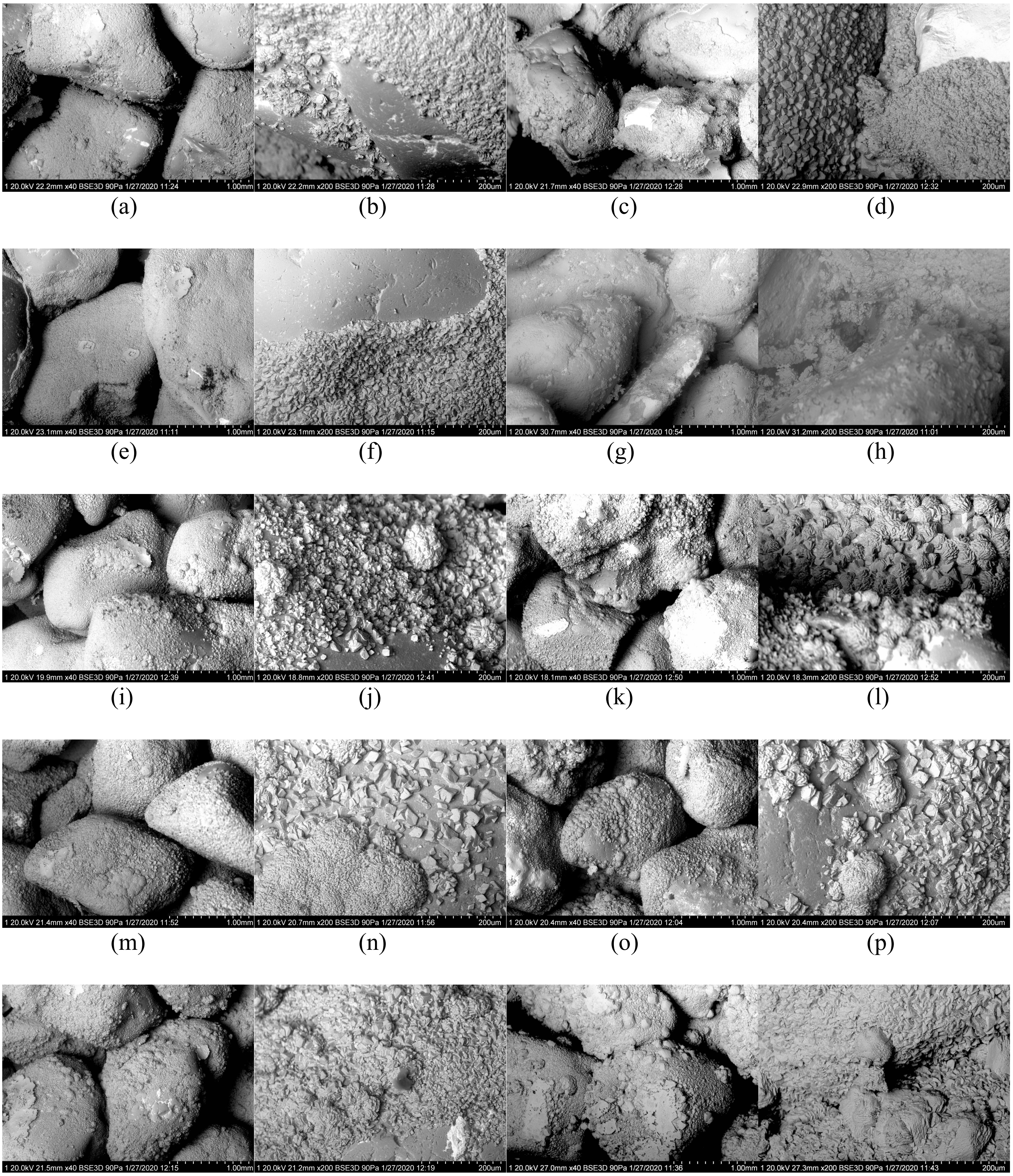

Figure S5. SEM imaging: each row represents the tests with one value of urease activity sorted from the largest to the smallest; the first two images in each row are for samples generated with OD_{600}=2 whilst the last two images of each row are for samples generated with OD_{600}=3. The lower magnifications show the position of the carbonate crystals relative to the grains and the larger magnifications focus on the carbonate crystal characteristics.

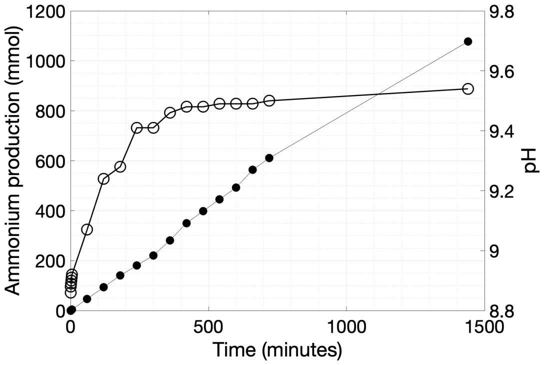

(a)

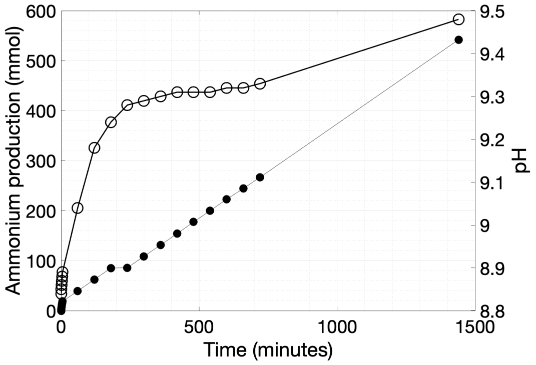

(b)

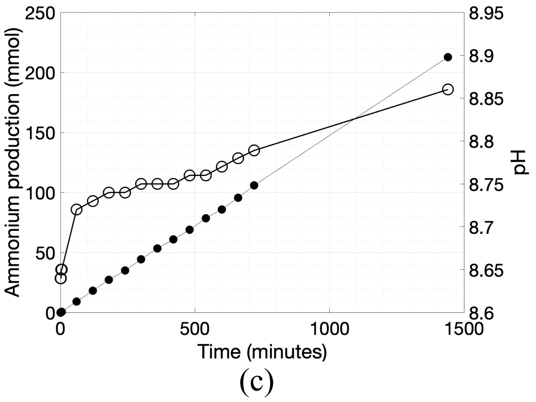

(c)

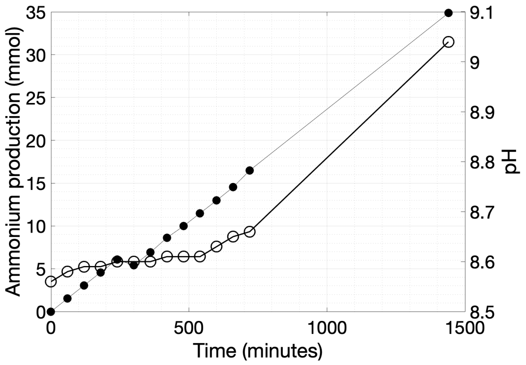

(d)

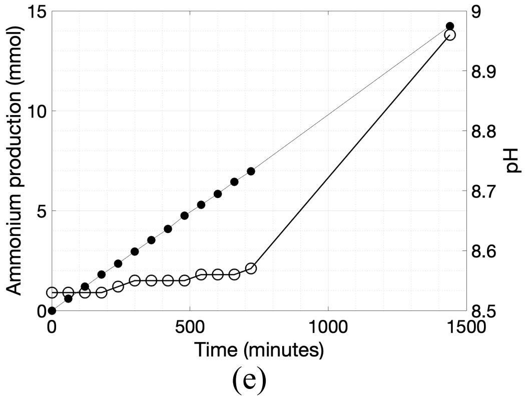

(e)

Figure S6. Ammonium production (mmol) measurements (●) and pH measurements (○) within a time window of 24 hours for the bacterial solutions utilised in the sand column experiments: (a) 237.4, (b) 112.9, (c) 44.1, (d) 7.5, and (e) 0.45 mmol/L/h measured following the protocol by Whiffin (2004).

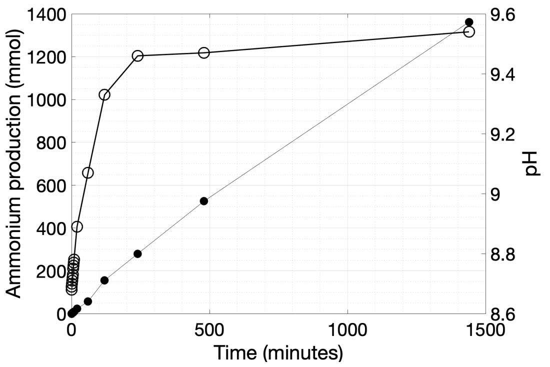

(a)

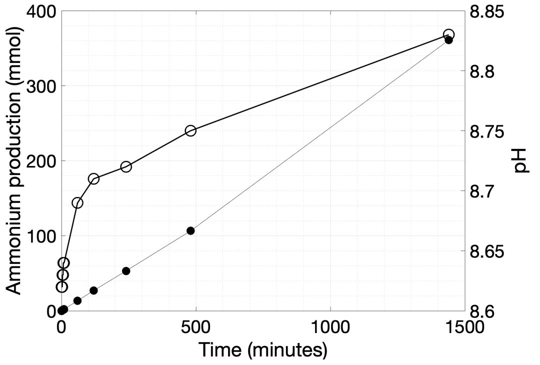

(b)

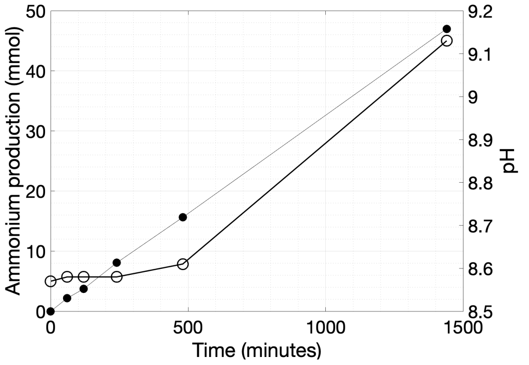

(c)

Figure S7. Ammonium production (mmol) measurements (●) and pH measurements (○) within a time window of 24 hours for the bacterial solutions utilised in the conical flask experiments: (a) 294.9, (b) 66.1 and, (c) 9.8 mmol/L/h measured following the protocol by Whiffin (2004).

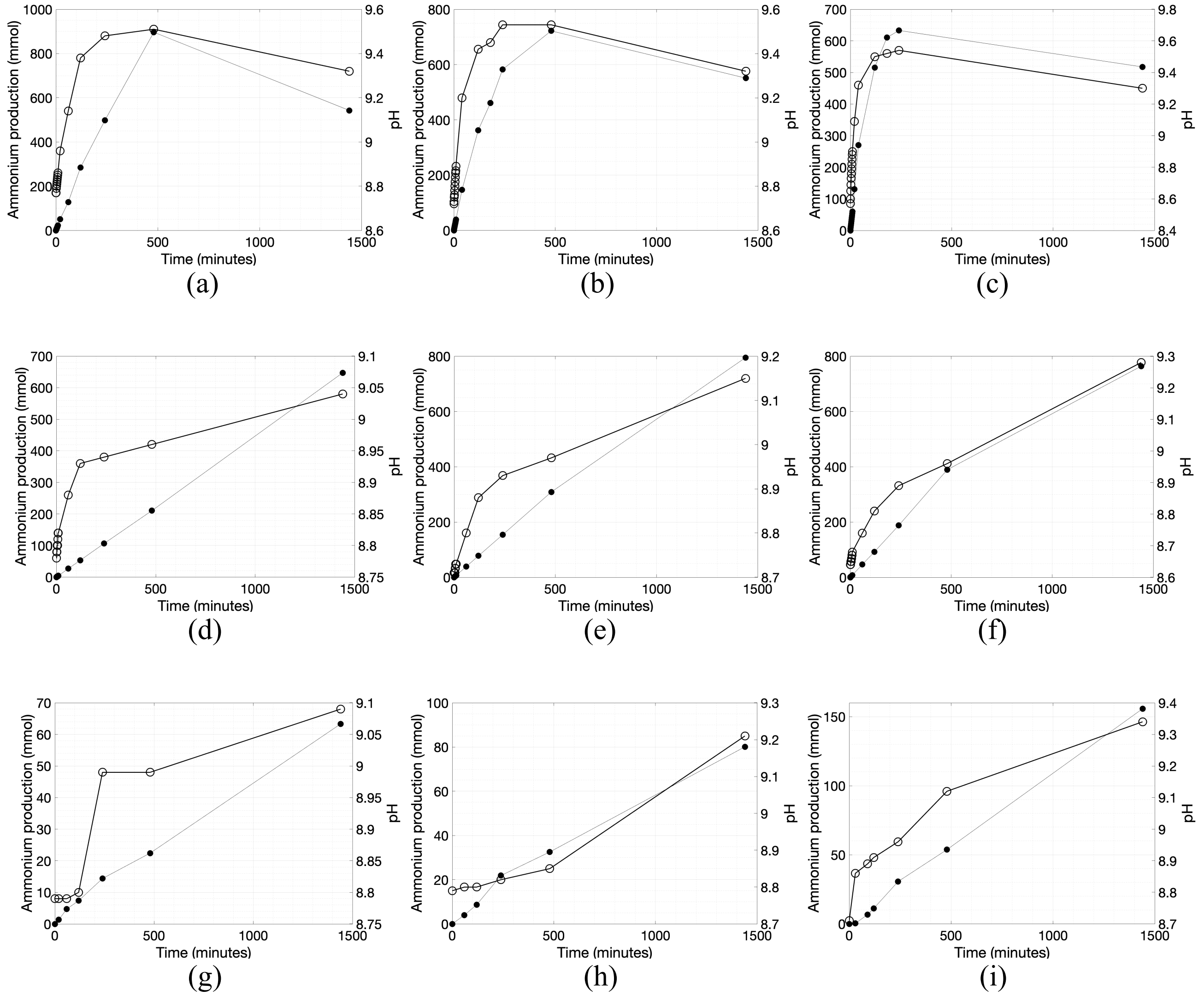

Figure S8. Ammonium production (mmol) measurements (●) and pH measurements (○) within a time window of 24 hours for the bacterial solutions utilised in the conical flask experiments measured following the protocol that replicates the experimental conditions (a) OD_{600}=1, 149.9 mmol/L/h, (b) OD_{600}=2, 231.9 mmol/L/h, (c) OD_{600}=3, 355.5 mmol/L/h, (d) OD_{600}=1, 26.5 mmol/L/h, (e) OD_{600}=2, 38.2 mmol/L/h, (f) OD_{600}=3, 46.1 mmol/L/h, (g) OD_{600}=1, 2.8 mmol/L/h, (h) OD_{600}=2, 4.7 mmol/L/h and, (i) OD_{600}=3, 6.9 mmol/L/h

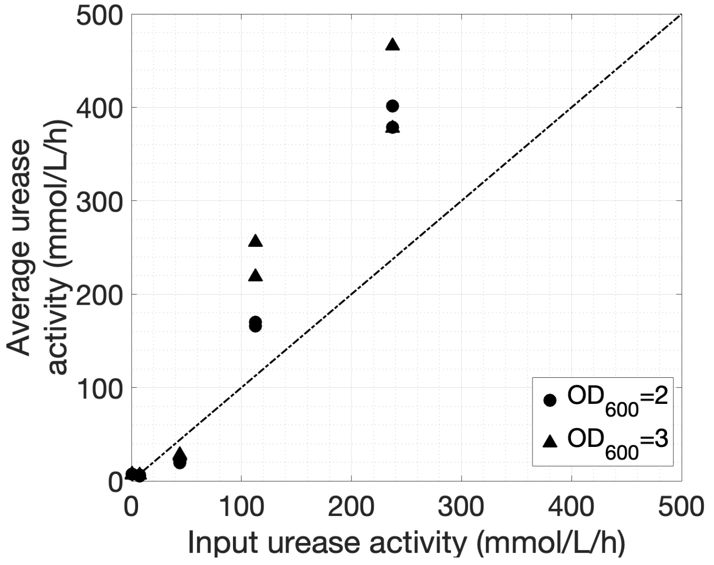

(a)

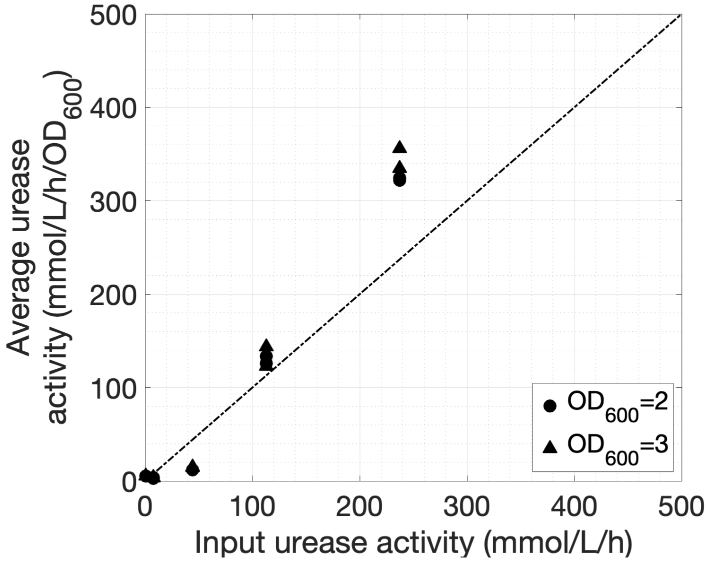

(b)

Figure S9. (a) Average output urease activity and (b) Average output specific urease activity with respect to the measured activity of the bacterial population before the injection.

Figure S9 shows the urease activity and the specific urease activity for each combination of bacterial population with respect to the input urease activity measured with the conventional method at a bacterial population of OD_{600}=1 for each specimen. The bacterial urease activity in the effluent was significantly different from the activity of the bacterial suspension before injection, as the measurements deviate substantially from the 1:1 line as shown in Fig. S8 (a). However, the differences in the values of activity in the injected and removed bacterial suspension remained consistent as shown in Fig. 8S (b), since there was no cross-over across the decreasing order of activities measured before the injection. The output urease activities had larger values compared to the input values. The specific urease activity of the effluent in the two cases with OD_{600}=2 and OD_{600}=3 was at the same levels, while the urease activity of the effluent was higher for OD_{600}=3 in the cases with the three highest activities. For the two experiments including the bacteria with the lowest urease activities there were no considerable differences. The activity measurements before and after the injection prove that indeed the pre-injection activity can be used as a reference value for MICP experiments.
